# Reversible cooperative dihydrogen binding and transfer with a bis-phosphenium complex of chromium†

**DOI:** 10.1039/d0sc03773g

**Published:** 2020-08-21

**Authors:** Nicholas Birchall, Christoph M. Feil, Michael Gediga, Martin Nieger, Dietrich Gudat

**Affiliations:** Institute of Inorganic Chemistry, University of Stuttgart Pfaffenwaldring 55 70550 Stuttgart Germany gudat@iac.uni-stuttgart.de; Department of Chemistry P.O. Box 55 00014 University of Helsinki Finland

## Abstract

The reversible reaction of H_2_ with a bis-phosphenium complex of chromium provides a rare example of 3d transition metal/phosphenium cooperativity. Photolysis induces the activation of H_2_ and yields a spectroscopically detectable phosphenium-stabilized (σ–H_2_)-complex, readily showing exchange with gaseous H_2_ and D_2_. Further reaction of this complex affords a phosphine-functionalized metal hydride, representing a unique example of reversible H_2_ cleavage across a 3d M=P bond. The same species is also accessible *via* stepwise H^+^/H^−^ transfer to the bis-phosphenium complex, and releases H_2_ upon heating or irradiation. Dihydrogen transfer from the H_2_-complex to styrene is exploited to demonstrate the first example of promoting hydrogenation with a phosphenium complex.

## Abstract 2

Photolysis of a phosphenium complex enables reversible activation of H_2_ to yield a dihydrogen complex which stimulates H_2_ cleavage or catalytic hydrogenation.

## Graphical abstract

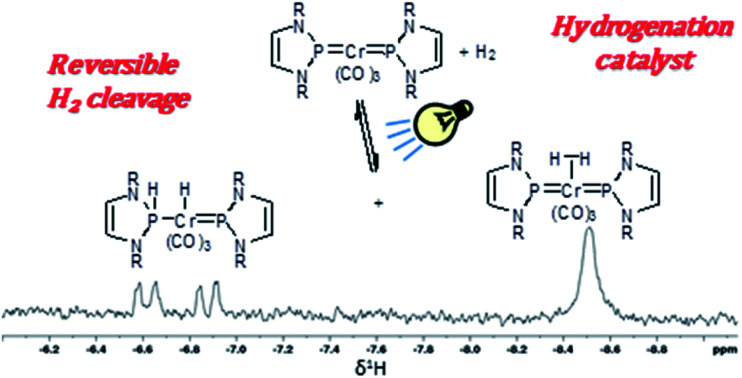

## Introduction

Cooperative metal ligand reactivity, implying that both the metal and a ligand of a transition metal complex participate in a bond activation process, has emerged as a new concept in homogeneous organometallic catalysis.^1^ Classical examples for the utilization of such metal/ligand cooperativity (MLC) are Noyori's^2,3^ approach to transfer-hydrogenation with amine/amide ruthenium complexes and Milstein's^4^ introduction of pyridine-based PNP-pincer ligands to the field. More recently, it was pointed out that the implementation of MLC through functional pincer ligands has in particular stimulated developing (de)hydrogenation reactions which use complexes of 3d metals as catalysts.^5^

**Scheme 1:**
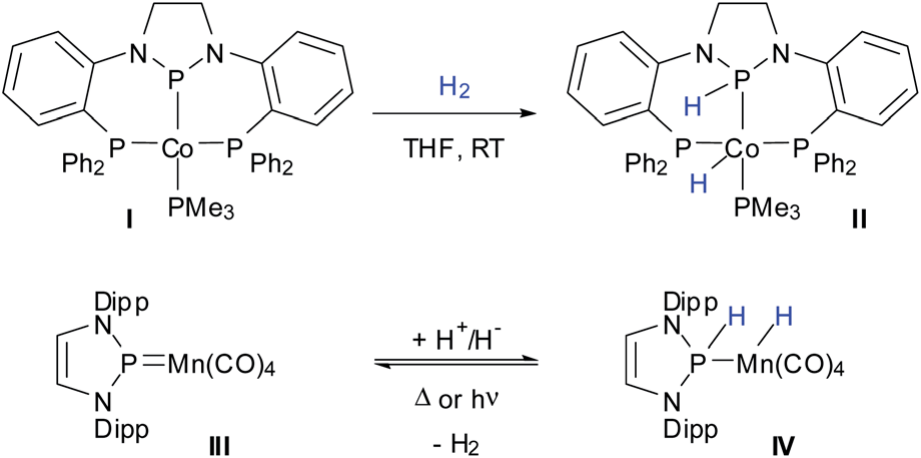
Reported examples of cooperative hydrogenation of metal–phosphorus bonds in complexes of 3d-transition metals (R = 2,6-iPr_2_C_6_H_3_).

Growing interest in the advance of MLC has inspired activities that are not only directed at rationally improving catalyst design, but also at searching for new combinations of cooperating ligands and 3d metals. While cooperative hydrogen activation using N–M bonds is well known,^1,5^ H_2_ addition across a P–M bond was long confined to rare cases involving second and third row transition metals.^6^ Only recently, the group of Thomas discovered the 1,2-addition of H_2_ across the covalent Co–P bond of a PPP-pincer complex **I** (Scheme 1) as the first example for cooperative activation of H_2_ on a P–M bond engrossing a 3d-metal.^7^ However, complex **II** proved inactive in reactions involving hydrogen transfer to other substrates, isotope exchange, or release of H_2_. To the best of our knowledge, reversible cooperative hydrogen activation on a P–M bond including a first row transition metal remains still unknown.

We had recently reported on the transformation of an N-heterocyclic phosphenium complex **III** into a phosphine-complex **IV** through stepwise addition of H^+^/H^−^ to a Mn=P bond (Scheme 1).^8^ Even if straight addition of H_2_ across the double bond was not accomplished, the observation of the reverse reaction, *viz.* thermally or photochemically induced dehydrogenation of **IV** to afford **III** and H_2_, implies that the forward transformation should in principle be feasible as well. Recollecting the potential redox non-innocence of the N-heterocyclic N_2_P-donor units in **I** and **III**,^9^ which is expressed in invoking in both cases identical limiting descriptions as either anionic N-heterocyclic phosphido (NHP^−^) or cationic N-heterocyclic phosphenium (NHP^+^) moieties, the complexes are electronically quite similar. If one considers further that **II** is likewise accessible from **I** through H^+^/H^−^ transfer from ammonia borane,^7^ the formation of **IV** and the hydrogenolysis of **I** can then be viewed as being closely related.

In view of the fact that MLC in diaminophosphenium complexes had already been established for cycloaddition and E–H-bond activation reactions,^10^ further exploration of the chemistry of these species is deemed a promising strategy in search of a viable route to cooperative hydrogenation of a bond between a phosphorus atom and a base metal. Along these lines, we investigated the reactivity of a bis-phosphenium complex of chromium towards H_2_ and report here on the first reversible addition of H_2_ across a double bond between phosphorus and a 3d-metal. This reaction involves a photochemically assisted activation step representing an unprecedented mechanism in the chemistry of phosphorus-based ligands. Moreover, we demonstrate that transfer of the ingested H_2_ molecule to a different substrate is feasible and enables hydrogenation of an olefin.

## Results and discussion

**Scheme 2:**
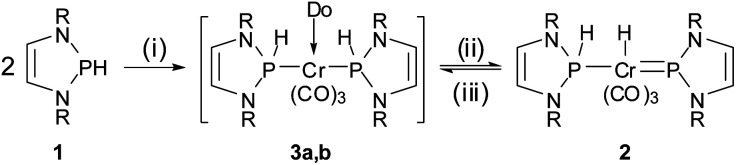
Synthesis of complex **2**. Reagents and conditions: (i) [Cr(CO)_3_(L)], THF, 16 h at r.t., – L; (ii) −Do; (iii) +Do (MeCN) (R = 2,6-iPr_2_C_6_H_3_, L = naphthalene, Do = THF (**3a**), MeCN (**3b**)).

**Scheme 3:**
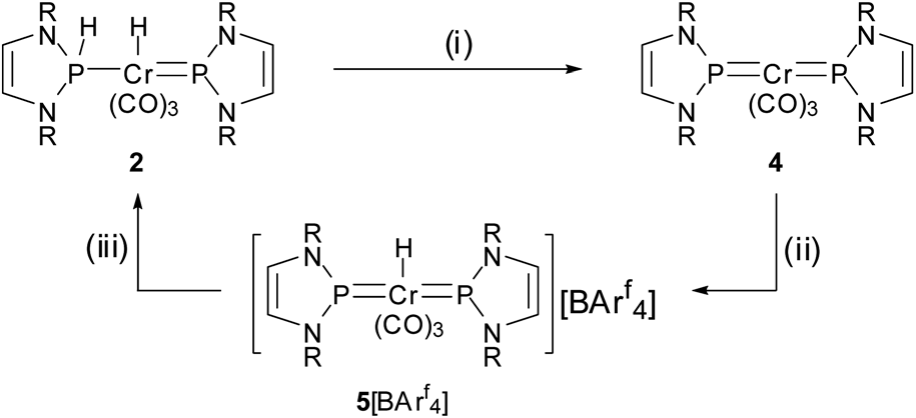
Interconversion between **2** and **4** by H^+^/H^−^ transfer and cooperative dehydrogenation. Reagents and conditions: (i) 12 h 80 °C, C_6_D_6_, –H_2_; (ii) ([H(OEt_2_)_2_)][BAr^f^_4_], C_6_H_6_, – 2 Et_2_O; (iii) Li[BEt_3_H], C_6_H_6_, 30 min, – Li[BAr^f^_4_], – BEt_3_ (R = 2,6-iPr_2_C_6_H_3_, Ar^f^ = 3,5-C_6_H_3_(CF_3_)_2_).

Our entry point to this chemistry was the synthesis of complex **2** (Scheme 2), representing the formal hydrogenation product of known bis-phosphenium complex **4** (ref. 11) (see Scheme 3), from [Cr(CO)_3_(naphthalene)] and two equivalents of secondary diazaphospholene **1** in THF. The product gives rise to two ^31^P NMR signals indicating the presence of NHP^+^ (*δ*^31^P 232 ppm) and secondary phosphine (*δ*^31^P 141 ppm, ^1^*J*_PH_ = 341 Hz) moieties. In accord with this assignment, the ^1^H NMR spectrum displays the expected signals attributable to phosphorus (*δ*^1^H 8.86) and metal bound (*δ*^1^H −6.34) hydrogens. We presume that the reaction is initiated by exchange of the coordinated arene in the metal precursor by THF to yield highly reactive [Cr(CO)_3_(THF)_3_],^12^ which is then converted into a transient bis-phosphine-complex **3a**. Formation of **2** is finally completed by 1,2-H-shift under dissociation of the last THF ligand.

**Fig. 1:**
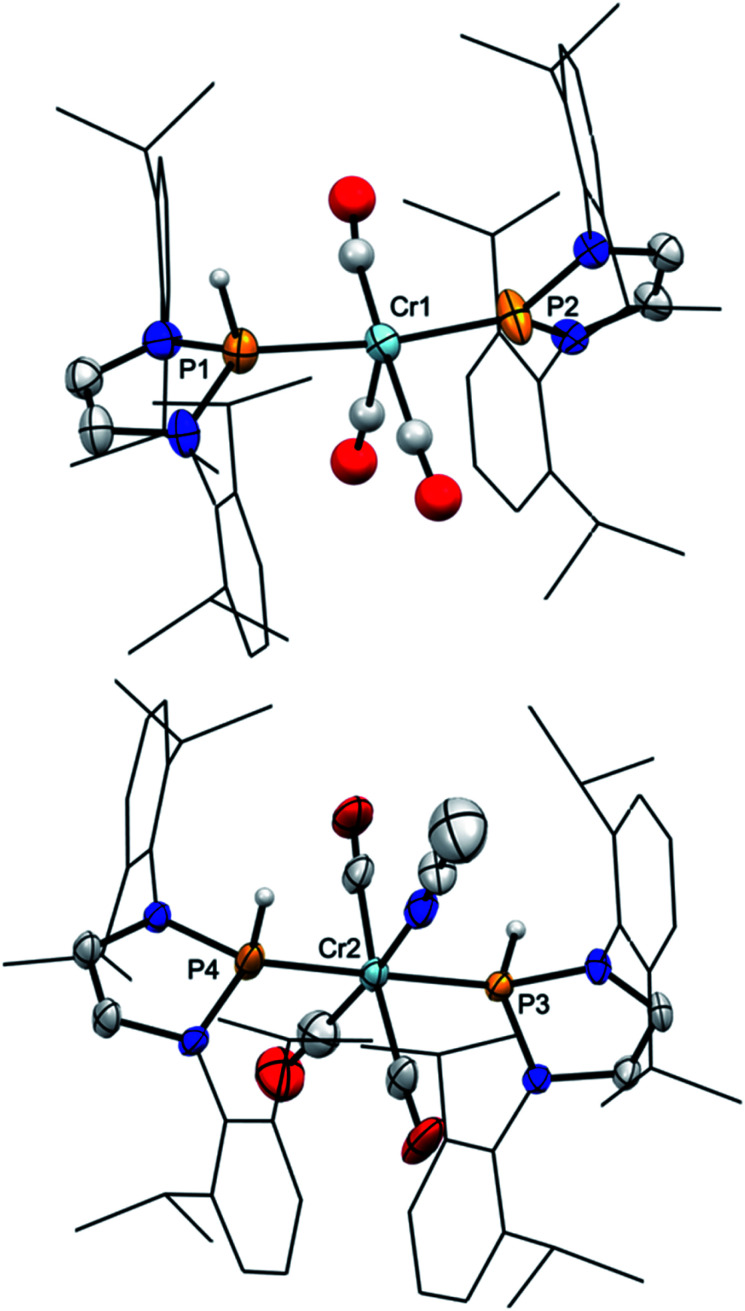
Molecular structure of **2** (top) and **3b** (bottom) in the crystal. Carbon-bound hydrogen atoms were omitted for clarity and Dipp-substituents were displayed using a wire model. Thermal ellipsoids were drawn at the 50% probability level. For **2**, only one of four possible orientations of the disordered part of the molecule is shown and the metal-bound hydrogen atom (the position of which could not be refined) is missing but the position of both hydrogen atoms is ascertained by spectroscopic data (see ESI for details†). Selected interatomic distances [Å]: **2**: Cr1–P1 2.224(2), Cr1–P2 2.150(2); **3b**: Cr2–P3 2.241(2), Cr2–P4 2.225(2). Sum of X–P–Y angles involving heavy atoms [°]: **2**: P1 344.2(2), P2 358.3(4); **3b**: P3 339.9(2), P4 345.1(2). “Envelope” fold angles along the N–N vectors of C_2_N_2_P-rings [°]: **2**: P2 8.4(3), P1 20.1(3); **3b**: P4 23.3(3), P3 17.3(4).

Experimental support for the proposed rearrangement was first obtained from ^1^H-EXSY NMR spectra, revealing reversible chemical exchange between metal- and phosphorus-bound hydrogens on a sub-second time scale. Moreover, titrating a solution of **2** in C_6_D_6_ with CD_3_CN afforded dynamic equilibrium mixtures in which both **2** and **3b** could be directly observed by NMR spectroscopy. The assignment of **3b** as bis-phosphine complex grounds on the observation of a single ^31^P NMR signal (*δ*^31^P 156.7) and the AA′XX′-type splitting of the resonance of the P-bound hydrogen atoms. Confirmation was found in an X-ray diffraction study of a single crystal, which had separated serendipitously from an equilibrated solution in C_6_H_6_/CH_3_CN (1 : 3.5) and contained a 1 : 1 mixture of **2** and **3b**. Complex **3b** (Fig. 1, bottom) features two phosphine ligands with P–Cr distances (Cr2–P3 2.241(2), Cr2–P4 2.225(2) Å) ranging at the lower end of known bond lengths in comparable chromium phosphine complexes (Cr–P 2.313(62) Å‡‡Median and standard deviation returned by a query in the CSD database for chromium complexes containing ligands of type PRY_2_ (R = any non-metal substituent, Y = O- or N-based substituent).) and five-membered rings adopting flat envelope conformation. Hydride complex **2** (Fig. 1, top) contains only one phosphine ligand (tetrahedral coordination at phosphorus, envelope-shaped ring, Cr1–P1 2.224(2) Å), while the second phosphorus atom exhibits the planar coordination environment and shortened P–Cr distance (Cr1–P2 2.150(2) Å) that are deemed characteristic for carbene-analogous NHP^+^ complexes.^13^

Having both compounds **2** and **4** (ref. 11) in hand, we were curious on studying their mutual interconversion by cooperative (de)hydrogenation (Scheme 3).

**Fig. 2:**
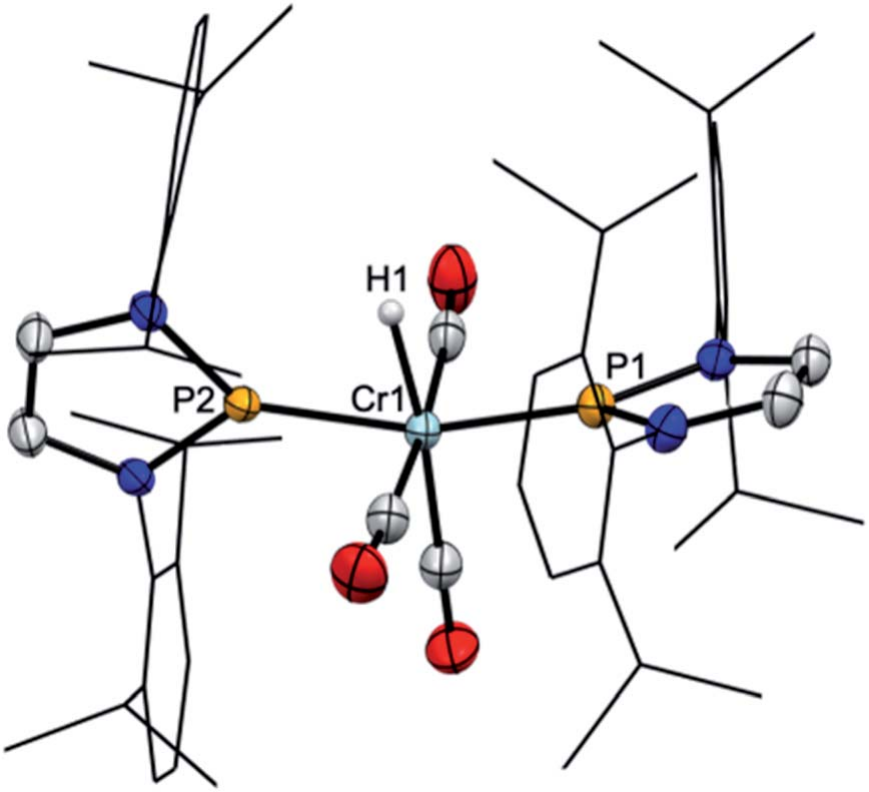
Molecular structure of the cation of **5**[BAr^f^_4_] in the crystal. Carbon-bound hydrogens were omitted for clarity. Dipp-substituents were displayed using a wire model. Thermal ellipsoids were drawn at the 50% probability level. Selected interatomic distances [Å]: Cr1–P2 2.135(1), Cr1–P3 2.138(1), Cr1–H1 1.632(23); sum of X–P–Y angles [°]: P2 359.7(1), P3 359.9(1).

Starting from **2**, we found that thermolysis at 80 °C resulted in evolution of H_2_ and formation of bis-phosphenium complex **4** as the only phosphorus-containing product detectable by ^31^P NMR spectroscopy. The reverse reaction was readily achieved in two steps through initial protonation of **4** with [H(OEt_2_)_2_][BAr^f^_4_] (Ar^f^ = 3,5-C_6_H_3_(CF_3_)_2_) to afford cationic bis-phosphenium complex **5**^+^, and subsequent treatment with super hydride.§§The order of addition is important; adding the reagents in reverse order gave in this case no conclusive results. Even if the order of both steps differs from that employed for conversion of **III** into **IV**,^8^ these reactions confirm that **4** exhibits, like mono-phosphenium complex **III**,^8^ nucleophilic character at the metal and electrophilic character on the NHP^+^ ligands. Phosphenium complex **5**[BAr^f^_4_] was isolated in crystalline form and unmistakably identified by its deshielded ^31^P NMR signal (*δ*^31^P 261, Δ*δ*^31^P +21 ppm *vs.***4**), the typical ^1^H NMR signal of the metal bound hydride (*δ*^1^H −7.9), and a single-crystal X-ray diffraction study. Cation **5**^+^ (Fig. 2) features two NHP moieties adopting a carbene-like coordination mode^9*b*,13^ distinguished by a planar coordination environment at the phosphorus atoms and short P–Cr distances (P1–Cr 2.135(1) Å, P2–Cr 2.138(1) Å).

The observation of a formal addition of H_2_ across the P=Cr bond of complex **4** by stepwise transfer of a H^+^/H^−^ pair brought up the question of whether the same product was accessible by direct activation of dihydrogen. We therefore studied the reactivity of **4** with H_2_ by monitoring the NMR spectra of solutions of the complex in THF-[D_8_] under H_2_-atmosphere (1 to 8 bar). While no reaction was observed at ambient temperature, the spectrum of a solution that had been heated for 300 h at 60 °C under 8 bar of H_2_ revealed indeed the formation of trace amounts of **2**. This finding confirms that direct hydrogenolyis of the bis-phosphenium complex is indeed in principle feasible, but rather ineffective under the conditions chosen.

**Fig. 3:**
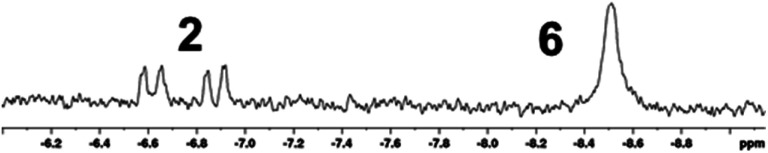
Expansion of the hydride region of the ^1^H NMR spectrum of a 14 mM solution of **4** in THF-[D_8_] after 7.5 h photolysis under 8 bar of H_2_ with signal assignment.

More promising results were obtained by performing the reaction under irradiation with a medium-pressure Hg-lamp. Inspection of the ^1^H NMR spectrum of a solution of **4** recorded after 7.5 h of photolysis under H_2_ (8 bar) revealed two signals with negative chemical shifts indicative of the formation of two new metal hydrides (Fig. 3).

Further analysis of 1D and 2D NMR spectra allowed us to assign one of these resonances to hydrogenation product **2** and to identify the second one as belonging to an isomeric complex featuring two equivalent NHP^+^ ligands (*δ*^31^P 234 ppm) and two metal-bound hydrogen atoms. Integration of suitable ^1^H NMR signals indicated that 20% of bis-phosphenium complex **4** had been converted to **2** and 16% to the yet unknown second hydrogenation product. Extended irradiation led to growing in of additional resonances, which we attribute to decomposition products, but did not affect further significant changes in the distribution of the main products, suggesting that a photo-stationary state had been reached.

**Scheme 4:**
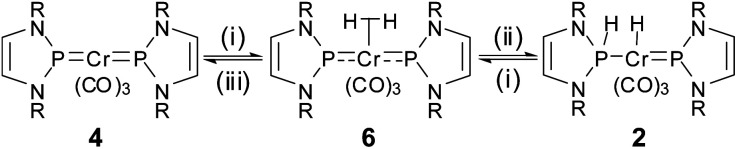
Cooperative hydrogenation of **4**. Reagents and conditions: (i) 8 bar H_2_, *hν*; (ii) 8 bar H_2_, 40 °C; (iii) vacuum 40 °C, several days.

Studies aiming at a more comprehensive description of the unknown hydrogenation product revealed that the hydride signal displays a very short *T*_1_ relaxation time (*T*_1min_ = 18 ms at 253 K), which is characteristic of dihydrogen complexes^14^ and led us to assign this product tentatively as (σ–H_2_)-complex **6** (Scheme 4). Photochemical generation of H_2_-complexes of chromium has precedence,^15^ and we could indeed confirm our initial assignment by isotope labelling studies. While the formation of specifically deuterated **2**-[D_2_] and **6**-[D_2_] during photolysis of **4** under D_2_-atmosphere proved in the first place merely the uptake of a hydrogen molecule from the gas phase, crucial structural information was gained from an irradiation experiment that was conducted with a H_2_/HD/D_2_-mixture and afforded a mix of all three possible isotopomers of **6**.

Analysis of the hydride ^1^H NMR signal of **6**-[D_1_] allowed us to determine a value of ^1^*J*_HD_ = 31.8 Hz and, using the well-established relation between ^1^*J*_HD_ and H–H distance (*d*_HH_ = 1.42 − 0.0167 × *J*_HD_^16^), to calculate *d*_HH_ = 0.89 Å. Both the values of ^1^*J*_HD_ and *d*_HH_ for **6** come close to reported data for H_2_-complexes of chromium.^15^

NMR spectroscopic monitoring of the photolysis over time revealed that formation of **6** precedes that of **2** and that the steady-state molar fractions of both species grow with increasing H_2_-pressure (see ESI†). Tempering reaction mixtures at 40 °C under 8 bar of H_2_ for several days without irradiation led to an eventual increase of the signals of **2** at the expense of those of **6**. As a corollary of these findings, we consider **6** as an intermediate in the formation of **2**. It should be noted that initial bonding and activation of a H_2_ molecule on the metal centre had also been postulated for the hydrogenolysis of **I**.^7^

Once formed, complex **6** was surprisingly thermally stable, showing no release of H_2_ within 18 h at 20 °C in an atmosphere of argon (1 bar), and decaying only slowly upon tempering the reaction mixture in a previously evacuated NMR tube at 40 °C for several days. The observed recovery of bis-phosphenium complex **4** and H_2_ under these conditions implies that the interchange between **4** and **6** under uptake or release of H_2_ is not coupled to (de)carbonylation, as in other cases.^15^ Contrasting its inert behaviour in the absence of external H_2_, complex **6** reacted *via* rapid incorporation of D_2_ and release of H_2_ upon exposure to a D_2_-atmosphere. The failure to detect any HD or **6**-[D_1_] suggests that the reaction proceeds, as in other cases,^15^ by exchange of intact H_2_/D_2_ molecules. We presume that the isomerization **6** → **2** under H_2_-pressure, which contrasts the slow dehydrogenation observed in the absence of a significant partial pressure of H_2_, is most likely a bimolecular process.

**Fig. 4:**
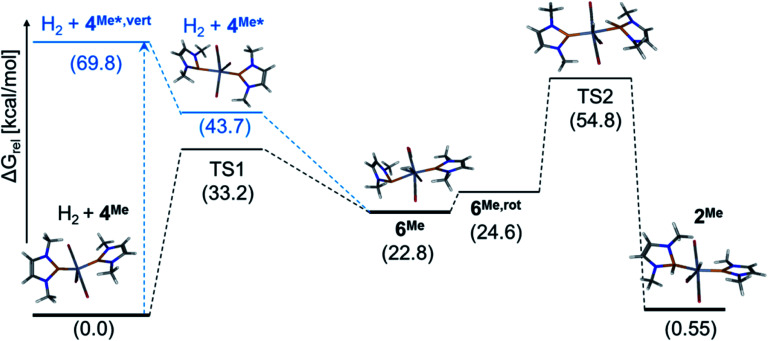
Computed free energy profile for the reaction of **4Me** with H_2_ and wire-model representations of the molecular structures of calculated stationary states. Relative free energies Δ*G*^0^ (in kcal mol^−1^) of electronic ground states (in black) and excited states (in blue) were obtained from DFT or TD-DFT calculations at the ωB97xD/def2-tzvp-level. 4Me*,vert and 4Me* denote the energies of the first excited state of **4Me** after vertical electron excitation and subsequent structural relaxation (calculated at the ωB97xD/def2-tzvp//ωB97xD/def2-svp-level), respectively. **6Me,rot** refers to a conformer obtained by rotation of the pyramidal NHP unit in **6Me**.

In order to gain mechanistic understanding of the hydrogen activation on **4**, we performed preliminary computational studies (at the ωB97xD/def2-tzvp-level of theory that had also been used to model similar reactions)^8^ on the hydrogenolysis of NMe-substituted complex **4Me** (see ESI for details†). The energy optimized molecular structures of **4Me** and **2Me** feature, in accord with the experimental findings on **4** and **2**, NHP^+^ units with planar coordinated phosphorus atoms and short P–Cr bonds (P–Cr 2.076–2.077 Å) with partial multiple bond character. The formal addition of H_2_ across a Cr=P bond of **4Me** is predicted to be faintly endergonic (Δ*G*^0^ = 0.6 kcal mol^−1^) but impeded by a high kinetic barrier (Δ*G*^#^TS2) = 54.8 kcal mol^−1^, (see Fig. 4). The computed molecular structure of dihydrogen complex **6Me** has two structurally different NHP units, one of which adopts the same “carbene-like” binding mode as in **2Me** and **4Me**, while the other one exhibits a pyramidal coordination at phosphorus and an elongated Cr–P distance (P–Cr 2.635 Å). Conceptually, this bonding mode can be associated with a description of the NHP unit either as an 1e-ligand (assuming covalent P–Cr-bonding^13^) or an anionic phosphide moiety^9,13*c*^ with a P-centred lone-pair of electrons that does not interact with the metal. This view is supported by an NBO analysis,^17^ which supposes the presence of a lone-pair and a reduced partial charge (*q*(NHP) = +0.38 *vs.* 1.28 for the planar NHP ligand) on the pyramidal NHP unit. Similar shifts in coordination modes have been previously associated with redox-non-innocent behaviour of phosphenium ligands and were considered to reflect a close relation to the chemistry of nitrosyls.^9^ The manifestation of two unlike NHP ligands contradicts at first glance the experimental data observed for **6**, but we located a low-lying (Δ*G*_rel_ = +4.4 kcal mol^−1^) excited singlet state with two indistinguishable NHP ligands that may provide for facile dynamic equilibration of both binding modes. The open shell nature and low electronic excitation energy of the excited singlet (Δ*E* = 7.5 kcal mol^−1^) and the likewise symmetrical triplet state (Δ*E* = 8.5 kcal mol^−1^) suggest that the electronic structure of **6Me** may be more complex than anticipated from our preliminary computations. Hydrogenation of **4Me** to afford **6Me** is kinetically less disfavoured (Δ*G*^#^(TS1) = 33.2 kcal mol^−1^) than addition of H_2_ across a P=Cr bond but strongly endergonic (Δ*G*^0^ = 22.8 kcal mol^−1^), explaining our failure to access **6** in a thermal reaction.¶¶Preliminary DFT calculations suggest that formal replacement of NMe by NPh substituents exerts a marked energetic stabilization of the H_2_-complex (the energy gap between **6Ph** and **4Ph** + H_2_ computed at the ωB97xD/def2-tzvp//ωB97xD/def2-svp level of theory is by 9.1 kcal mol^−1^ lower than between the NMe-derivatives). The change is still way too small to render the hydrogenation exergonic, but highlights the qualitative nature of our computational model. The effect on **2Ph** (stabilized by 0.9 kcal mol^−1^ at the same level) is only minute.

A TD-DFT calculation on **4Me** allowed us to assign the energetically lowest electron excitation as a transition with MLCT-character that transfers electron density from the Cr-centred Kohn–Sham (KS) HOMO into the NHP-centred KS-LUMO (see ESI†). Energy optimization of the vertically excited state furnished a relaxed molecular geometry distinguished by one planar and one pyramidal NHP unit with similar characteristics as in **6Me** (Fig. 4). Adopting the notion of pyramidal and planar NHPs as 1e- and 3e-ligands,^13^ electronically excited 4Me* can be pictured as complex with a 16 VE count that should be capable of binding a further Lewis donor. In accord with this hypothesis, a relaxed potential energy scan revealed that reaction of 4Me* with H_2_ allows accessing **6Me** in an exergonic process without having to pass a further energy barrier. Additional computations implied that binding of a solvent (THF) is likewise feasible, but less exergonic compared to formation of **6Me** (see ESI†). The energetic ordering of TS1 and TS2, representing competing pathways for the unimolecular decay of **6Me**, is qualitatively in accord with the observed formation of H_2_ and **4** during thermolysis of **6**.

**Scheme 5:**
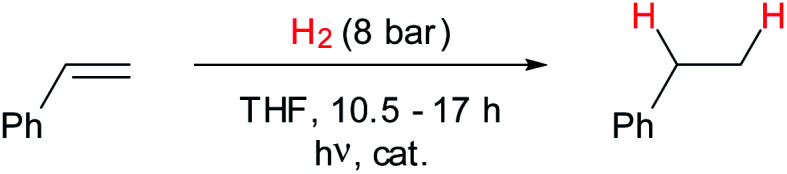
Photocatalytic hydrogenation of styrene.

The observed exchange with free dihydrogen brought up the question of whether complex **6** was also capable of transferring a H_2_-molecule to another substrate. Studying the photolysis of solutions of **4** in THF-[D_8_] under 8 bar of H_2_ in the presence of an excess of styrene, we noticed indeed an over-stoichiometric formation of ethylbenzene with up to 99% conversion (Scheme 5 and Table 1). Control experiments revealed that no hydrogenation took place in the absence of **4**, or when only phosphine **1** was present,||||This result allows excluding catalysis by phosphinyl radicals formed by photolysis of **1**; see ref. 18. whereas [Cr(CO)_3_(naphthalene)] gave a single turnover, which is in accord with a stoichiometric rather than catalytic reaction. The incomplete consumption of styrene with low initial complex loadings (Table 1, entry 2) is presumably due to eventual conversion of **4** and **6** into inactive follow-up products. In accord with this conjecture, NMR studies indicated formation of an alkyl phosphine arising from formal hydrophosphination of styrene, along with minor amounts of **1** and further unknown phosphorus-containing species as by-products of the hydrogenation. Analysing the composition of a reaction mixture that had been irradiated for 20 min and was then stored in the dark for 18 h at 20 °C revealed further that the signal of **6** generated during photolysis had disappeared, while **2** was still present, and an approximate 10-fold excess of ethylbenzene (in relation to the molar amount of **6** consumed) had newly formed.

**Table 1:** Results of the photocatalytic hydrogenation of styrene^a^

Entry	Catalyst	Mol%	Irradn. time [h]	Conv.^b^ [%]	TON^c^
1	**4**	16	11.5	99	6
2	**4**	4	10.5	76	18.5
3	**1**	16	17	0	0
4	[Cr(CO)_3_(L)]^d^	16	17	18	1.1
5	None	—	17	0	0

a Conditions: 26 μmol styrene in THF-[D_8_] (0.5 mL), 8 bar H_2_, irradiation with a medium-pressure Hg lamp.

b Conversion determined by integration of suitable ^1^H NMR signals.

c TON = *n*(C_6_H_5_Et)/*n*(complex).

d L = naphthalene.

We interpret these findings as confirmation that H_2_-complex **6** is the active species in promoting olefin hydrogenation, while **2** acts merely as spectator. The discovery that complex **6** is obviously capable of accomplishing multiple turnovers without further photochemical activation is in accord with a catalytic mechanism. It is worthwhile mentioning that the observed hydrogenation of styrene, even if its performance cannot yet compete with established schemes for catalytic hydrogenation and requires further optimization, provides nonetheless first a proof of concept for the feasibility of using a phosphenium base–metal complex as hydrogenation catalyst.

## Conclusions

In summary, we have accomplished the first reversible cooperative addition of molecular H_2_ to a double bond between phosphorus and a first-row transition metal, and demonstrated further the transfer of the ingested H_2_-molecule in the hydrogenation of an olefin. Key to this reactivity is the stimulation of the initially present bis-N-heterocyclic phosphenium complex by photolysis, which paves the way to formation of a Kubas-type “non-classical” (σ–H_2_)-complex^19^ as crucial intermediate in both Cr=P hydrogenolysis and H_2_-transfer. The finding that activation of H_2_ occurs on the metal centre confirms earlier conjectures,^7^ and is also supported by computational studies. The observed reaction pathway is an unprecedented approach to using the specific reactivity of a P-donor ligand for generation of a vacant coordination site on an electronically saturated (18 VE) transition metal centre, even if it does not provide an example of metal–ligand cooperativity in a strict sense.^5^ Nonetheless, its feasibility relies crucially on the redox non-innocence of the phosphenium ligand and is inconceivable without cooperative interplay between metal and ligand. We are currently striving to improve the performance of NHP-complex-mediated hydrogenation and extend its application to further types of multiple bonds, and to explore the photolytic stimulation of NHP complexes as a more generally applicable tool for promoting metal binding and activation of other ligands than H_2_.

## Conflicts of interest

There are no conflicts to declare.

## Supplementary Material

SC-011-D0SC03773G-s001

SC-011-D0SC03773G-s002
