# Supplementary material for: Reversible cooperative dihydrogen binding and transfer with a bis-phosphenium complex of chromium
Source: Chem Sci. 2020 Aug 21;11(35):9571–6. doi: 10.1039/d0sc03773g (PMC8161683; doi:10.1039/d0sc03773g)
Supplement: SC-011-D0SC03773G-s001 [file SC-011-D0SC03773G-s001.pdf]

Electronic Supplementary Material (ESI) for Chemical Science.
This journal is © The Royal Society of Chemistry 2020

Supplementary information

Reversible Cooperative Dihydrogen Binding and Transfer with a Bis-Phosphenium Complex of Chromium

Nicholas Birchall, Christoph M. Feil, Michael Gediga, Martin Nieger and Dietrich Gudat

Institute of Inorganic Chemistry, University of Stuttgart, Stuttgart, Germany

E-Mail: dietrich.gudat@iac.uni-stuttgart.de

Table of contents

Experimental Procedures	p. 3
Syntheses	p. 3
Reactivity Studies	p. 4
NMR-Spectra	p. 17
High Resolution Mass Spectra	p. 24
UV-VIS Spectra	p. 24
Crystallographic Studies	p. 25
Computational Studies	p. 25

Experimental Procedures

Due to the air and moisture sensitivity of the studied compounds, all manipulations were carried out in flame-dried glassware under an inert gas atmosphere of purified argon. Experiments with H_2/D_2 were carried out in medium-walled valved NMR-Tubes (Norell® extreme series) under an atmosphere of purified gas. $Cr(CO)_3$(naphthalene),[1] $Na_2[Cr(CO)_5]$,[2] 2-chloro-1,3-bis(2,6-diisopropylphenyl)-1,3,2-diazaphospholene,[3] 2-hydrido-1,3-bis(2,6-diisopropylphenyl)-1,3,2-diazaphospholene[3] (**1**) and Brookhart's acid[4] were synthesized as described. THF, *n*-pentane, *n*-hexane, benzene and diethyl ether were distilled from NaK alloy and CH_2Cl_2 and CH_3CN from CaH_2 prior to use. C_6D_6 and THF-D_8 were refluxed over NaK alloy for 72 h and CD_3CN over CaH_2 for 48 h, followed by distillation and storage over molecular sieves in a glovebox. $LiHBEt_3$ (1.0 M solution in THF) was purchased from Sigma Aldrich and used without further purification. Irradiation experiments were carried out with a medium pressure mercury lamp. NMR spectra were acquired on Bruker Avance 250 (^{1}H: 250.0, ^{13}C: 62.9, ^{31}P: 161.9 MHz) or Bruker Avance 400 (^{1}H: 400.1 MHz, ^{13}C: 100.5 MHz, ^{31}P: 161.9 MHz) NMR spectrometers at 293 - 296 K if not stated otherwise. ^{1}H Chemical shifts were referenced to TMS using the signals of the residual protons of the deuterated solvent ($\delta^{1}H$ = 7.15 (C_6D_6), 1.73 (THF-D_8)) as secondary reference. NMR Spectra of heteronuclei were referenced using the Ξ-scale[5] with TMS (Ξ = 25.145020 MHz, ^{13}C) and 85 % H_3PO_4 (Ξ = 40.480747 MHz, ^{31}P) as secondary references. Carbon and hydrogen atoms in diazaphospholene and aromatic rings are labelled as NCH and *i*-C, *o*-C etc., respectively. FTIR spectra were measured on a Thermo Scientific/Nicolet iS5 instrument equipped with an iD5 attenuated total reflectance (ATR) accessory under N_2 atmosphere. Combustion analyses were performed on an Elementar Micro Cube elemental analyzer. Mass spectra were obtained with a Bruker Daltonics Microtof-Q mass spectrometer. UV/VIS spectra were recorded with a J&M TIDAS spectrophotometer.

Syntheses

Complex 2

(a) $[Cr(CO)_3$(naphthalene)] (130 mg, 0.50 mmol) and **1** (400 mg, 1.00 mmol) were dissolved in THF (10 mL). The solution was stirred for 16 h under exclusion of light. The solvent was removed under reduced pressure and the resulting solid suspended in *n*-pentane (35 mL, 30 min sonication with ultrasound to ensure complete dispersion). Filtration of the suspension over a frit (por. IV), washing the resulting fine orange powder with *n*-pentane (2 x 20 mL) and drying for 1 h in vacuum gave 250 mg (0.26 mmol, 54 %) of **2**.

^{1}H NMR (250 MHz, C_6D_6): δ = 8.86 (ddd, 1 H, $^{1}J_{PH}$ = 341 Hz, $^{3}J_{PH}$ = 6 Hz, $^{3}J_{HH}$ = 5.8 Hz, PH), 7.30-7.00 (m, 12 H, C_6H_3), 6.14 (d, 2 H, $^{3}J_{PH}$ = 5.9 Hz, NCH), 5.94 (d, 2 H, $^{3}J_{PH}$ = 6.3 Hz, NCH), 3.59 (sept, 2 H, $^{3}J_{HH}$ = 6.6 Hz, CH), 3.26 (sept, 2 H, $^{3}J_{HH}$ = 6.6 Hz, CH), 3.20 (sept, 4 H, $^{3}J_{HH}$ = 6.6 Hz, CH), 1.40 (d, 6 H, $^{3}J_{HH}$ = 6.6 Hz, CH_3), 1.35 (d, 6 H, $^{3}J_{HH}$ = 6.6 Hz, CH_3), 1.25 (d, 18 H, $^{3}J_{HH}$ = 6.6 Hz, CH_3), 1.17 (d, 6 H, $^{3}J_{HH}$ = 6.6 Hz, CH_3), 1.09 (d, 12 H, $^{3}J_{HH}$ = 6.6 Hz, CH_3), -6.34 (ddd, 1H, $^{2}J_{PH}$ = 105 Hz, $^{2}J_{PH}$ = 29 Hz, $^{3}J_{HH}$ = 5.5 Hz, CrH). – $^{13}C\{^{1}H\}$ NMR (62.9 MHz, C_6D_6): δ = 222 (br, CO), 149.9 (s, *o*-C), 149.8 (s, *o*-C), 147.5 (s, *o*-C), 138.5 (d, $^{2}J_{PC}$ = 4.6 Hz, *i*-C), 130.2 (s, *p*-C), 126.1 (s, NCH), 124.5 (s, *m*-C), 124.4 (s, *m*-C), 123.6 (s, *m*-C), 122.6 (s, NCH), 30.3 (s, CH), 29.2 (s, CH), 28.7 (s, CH), 27.3 (s, CH_3), 25.3 (s, CH_3), 25.1 (s, CH_3), 23.8 (s, CH_3), 23.6 (s, CH_3), 23.0 (s, CH_3). – $^{31}P\{^{1}H\}$ NMR (161.9 MHz, C_6D_6): δ = 232 (d, $^{2}J_{PP}$ = 12 Hz), 141 (d, $^{2}J_{PP}$ = 12 Hz). ^{31}P NMR (161.9 MHz, C_6D_6): δ = 232 (ddd, $^{2}J_{PP}$ = 12 Hz, $^{2}J_{PH}$ = 29 Hz, $^{3}J_{PH}$ = 6 Hz), 141 (ddd, $^{1}J_{PH}$ = 341 Hz, $^{2}J_{PH}$ = 107 Hz, $^{2}J_{PP}$ = 12 Hz). – IR (THF): ṽ = 1992 (s), 1906 (s), 1898 (s) cm^{-1}, ν(CO); 2160 (w) cm^{-1}, ν(PH). –$C_{55}H_{74}CrN_4O_3P_2$ (953.17 g mol^{-1}): calc. C 69.31 H 7.83 N 5.62, found C 68.03 H 7.84 N 5.50. – HRMS ((+)-ESI): *m/e =* 951.4554 ($[M-H]^+$, calcd. for $C_{55}H_{73}CrN_4O_3P_2$: 951.4557).

(b) $LiHBEt_3$ (105 µl 1 M solution in THF, 105 µmol) was added to a solution of **5**$[BAr^f_4]$ (100 µmol) in benzene (10 mL) that had been prepared as described below. The solution was stirred for 30 min at 25 °C. A slight fade in color to light orange was observed. The mixture was filtered over diatomaceous earth and the filtrate evaporated to dryness. A mixture of the residual orange solid with *n*-pentane (10 mL) was sonicated in an ultrasonic bath for 30 min. Filtration over a frit (por. IV) and washing the remaining orange powder with *n*-pentane (2 x 3 mL) gave analytically pure **2** (32 mg, 30 µmol, 31 %).

Complex 4

A slight optimization of the previously published protocol[6] was applied. A small amount of THF (0.5 mL) was added at room temperature to a stirred suspension prepared from $Na_2[Cr(CO)_5]$ (119 mg, 0.50 mmol) and 2-chloro-1,3-bis(2,6-diidopropylphenyl)-1,3,2-diazaphospholene (440 mg, 1.00 mmol) in *n*-hexane (10 mL). An immediate color change from clear brown to dark purple accompanied by vigorous gas evolution was observed. The solution was stirred for additional 30 min until gas formation had ceased. Volatiles were removed under reduced pressure and the resulting purple solid suspended in *n*-hexane (30 mL). Filtration over diatomaceous earth, concentration of the filtrate to approx. one fourth of the original volume, and storage at -15°C gave **2** as purple crystals (yield 340 mg, 0.35 mmol, 72 %).

A metallate-free synthesis of analytically pure **4** is feasible by stirring a solution of **2** (100 mg, 100 µmol) in CH_2Cl_2 (10 mL) for 18 h at 40 °C and subsequent removal of the solvent under reduced pressure (yield 75 mg, 75 µmol, 75 %).

^{1}H NMR (THF-D_8, 400.1 MHz): δ = 7.25-7.06 (m, 12 H, C_6H_3), 6.41 (pseudo-t, 4 H, NCH), 3.24 (sept, 8 H, $^{3}J_{HH}$ = 6.8 Hz, CH), 1.20 (d, 24 H, $^{3}J_{HH}$ = 6.8 Hz, CH_3), 1.12 (d, 24 H, $^{3}J_{HH}$ = 6.8 Hz, CH_3). – $^{13}C\{^{1}H\}$ NMR (62.9 MHz, C_6D_6): δ = 233 (br, CO), 147.6 (s, *o*-C), 136.5 (s, *i*-C), 129.4 (s, *p*-C), 125.1 (s, NCH), 124.6 (s, *m*-C), 29.1 (s, CH), 25.5 (s, CH_3), 23.6 (s, CH_3). – $^{31}P\{^{1}H\}$ NMR (THF-D_8): δ = 240.3 (s). IR (THF): ṽ = 1963 (s), 1934 (s), 1886 (s) cm^{-1}, ν(CO). – $C_{55}H_{74}CrN_4O_3P_2$ (951.15 g mol^{-1}): calcd. C 69.45 H 7.65 N 5.89, found C 68.73, H 7.65, N 5.71. – HRMS ((+)-ESI): *m/e =* 950.4476 ($[M-H]^+$, calcd. for $C_{55}H_{71}CrN_4O_3P_2$: 950.4479.

Complex 5[BAr$^f_4$]

A solution prepared from complex **4** (100 mg, 100 µmol) and [H(OEt$_2$)$_2$)][BAr$^f_4$] (106 mg, 100 µmol, Ar^f = $C_6H_3(CF_3)_2$) in benzene (10 mL) was stirred for 30 min at 25°C. A slow color change from purple to clear orange was observed. Storage at 4 °C gave analytically pure **5**[BAr$^f_4$] in crystalline form (96 mg, 53 µmol, 53 %).

^{1}H NMR (C_6D_6/THF-D_8 5:1, 400.1 MHz): δ = 8.22 (m, 8 H, *o*-Ar^f), 7.64 (m, 4 H, *p*-Ar^f), 7.16 (m, 4 H, C_6H_3), 7.00 (m, 8 H, C_6H_3), 6.91 (m, 4 H, NCH), 2.59 (sept, 8 H, $^3J_{HH}$ = 6.7 Hz, CH), 1.04 (d, 24 H, $^3J_{HH}$ = 6.7 Hz, CH_3), 0.95 (d, 24 H, $^3J_{HH}$ = 6.7 Hz), -7.94 (t, 1 H, $^2J_{PH}$ = 74.9 Hz). – ^{13}C NMR (C_6D_6/THF-D_8 5:1, 100.5 MHz): δ = 220.0 (br, CO), 160.0 (br, *i*-Ar^f), 145.9 (s, *o*-C), 135.2 (br, *o*-Ar^f), 131.7 (s, NCH), 129.2 (br, *m*-Ar^f), 128.1 (s, *p*-C), 125.1 (br q, $^1J_{CF}$ = 272 Hz, CF_3), 125.0 (s, *m*-C), 117.8 (br, *p*-Ar^f), 29.4 (s, CH), 24.7 (s, CH_3), 22.3 (s, CH_3). – ^{31}P{^{1}H} NMR (C_6D_6/THF-D_8 5:1, 161.9 MHz): δ = 259.7 (br). ^{31}P NMR (C_6D_6/THF-D_8, 5:1, 161.9 MHz): δ = 259.7 (d, $^2J_{PH}$ = 75 Hz). – IR (THF): ṽ = 2021 (s), 1981 (s), 1949 (s) cm^{-1}, ν(CO); 2082 (w) cm^{-1}, ν(CrH). HRMS ((+)-ESI): *m/e* = 951.4559 ([5$^+$], calcd. for $C_{55}H_{73}CrN_4O_3P_2$: 951.4557. – HRMS ((-)-ESI): *m/e* = 863.0648 ([BAr$^f_4$$^-$], calcd. for $C_{32}H_{12}BF_{24}$: 863.0643.

Characterization of Complex 3b and isolation of co-crystals of 2/3b.

For spectroscopic characterization of 3b, a solution of complex 2 (20 mg, 20 µmol) in CD_3CN/C_6D_6 (6:1, total volume 0.7 mL) was sonicated in an ultrasonic bath for 15 min. Analysis of the ^{1}H NMR spectrum indicated the presence of a 70:30 mixture of 3a and 2. An incomplete set of NMR data of 3b could be determined by analysis of 2D NMR spectra of the equilibrium mixture. Crystals containing an 1:1 mixture of both species suitable for a single-crystal X-ray diffraction study were grown from a solution of 2 (40 mg, 40 µmol) in toluene/MeCN (4:14, 18 mL total volume).

3b: ^{1}H NMR (CD_3CN/C_6D_6 6:1, 250 MHz): δ = 8.62 (AA'XX'-pattern, $^1J_{PH}$ = 277 Hz, $^3J_{PH}$ = 2.5 Hz, $^4J_{HH}$ = 2.5 Hz, $^2J_{PH}$ = 4.5 Hz, 2 H, PH), 7.46-7.07 (m, C_6H_3), 6.04 (m, 4 H, NCH), 3.77 (sept, 4 H, $^3J_{HH}$ = 6.8 Hz, CH), 3.28 (sept, 4 H, $^3J_{HH}$ = 6.8 Hz, CH), 1.27 (d, $^3J_{HH}$ = 6.8 Hz, CH_3), 1.23 (d, $^3J_{HH}$ = 6.8 Hz). – ^{13}C NMR (CD_3CN/C_6D_6 6:1, 62.9 MHz): δ = 228 (br, CO), 150.9 (s, *o*-C), 135.3 (s, *i*-C), 130.8 (s, *p*-C), 124.8 (s, *m*-C), 123.1 (s, NCH), 29.8 (s, CH), 29.4 (s, CH), 27.4 (s, CH_3), 25.1 (s, CH_3), 24.0 (s, CH_3), 23.6 (s, CH_3). – ^{31}P{^{1}H} NMR (CD_3CN/C_6D_6 6:1, 161.9 MHz) δ = 155.4 (s). ^{31}P NMR (CD_3CN/C_6D_6 6:1, 161.9 MHz) δ = 155.4 (br d, 277 Hz).

Reactivity Studies

Thermolysis of 2.

A solution of **2** (13 mg, 13 µmol) in C_6D_6 (0.5 mL) in a valved NMR tube was homogenized by sonification for 10 min and then tempered at 80 °C for 12 h. NMR spectra recorded before and after thermolysis revealed conversion of **2** into **4** and H_2 (Figure S1).

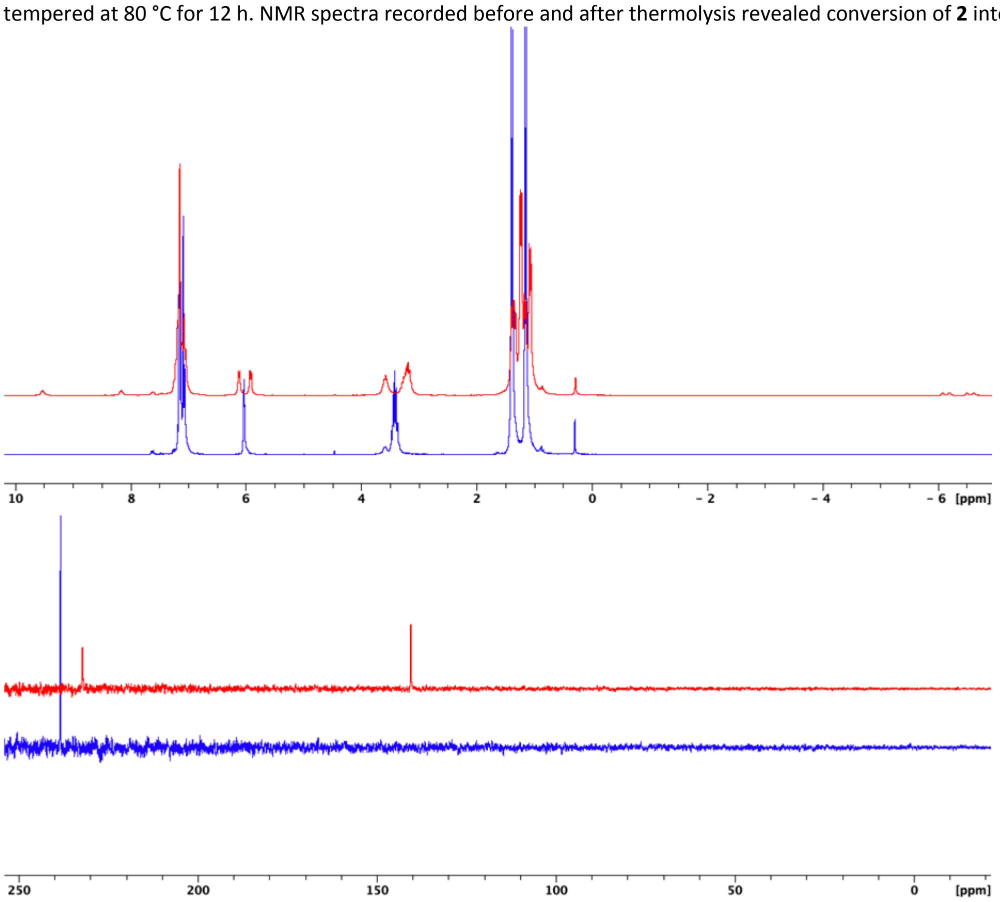

Figure S1. ^{1}H (top) and ^{31}P{^{1}H} NMR spectra recorded before (red traces) and after (blue traces) tempering a solution of **2** in C_6D_6 for 12 h at 80 °C. The ^{1}H NMR signal at 4.5 ppm is attributable to molecular H_2.

Thermal reaction of 4 with H_2

A solution of **4** (7 mg, 7 µmol) in THF-D_8 (0.5 mL) was transferred to a medium-walled high pressure NMR tube and degassed by completing three freeze-pump-thaw cycles. The NMR tube was then pressurized with H_2 (8 bar) and kept at 60° C for 300h. NMR spectra recorded before and after heating showed that trace amounts of **2** had formed (Figure S2).

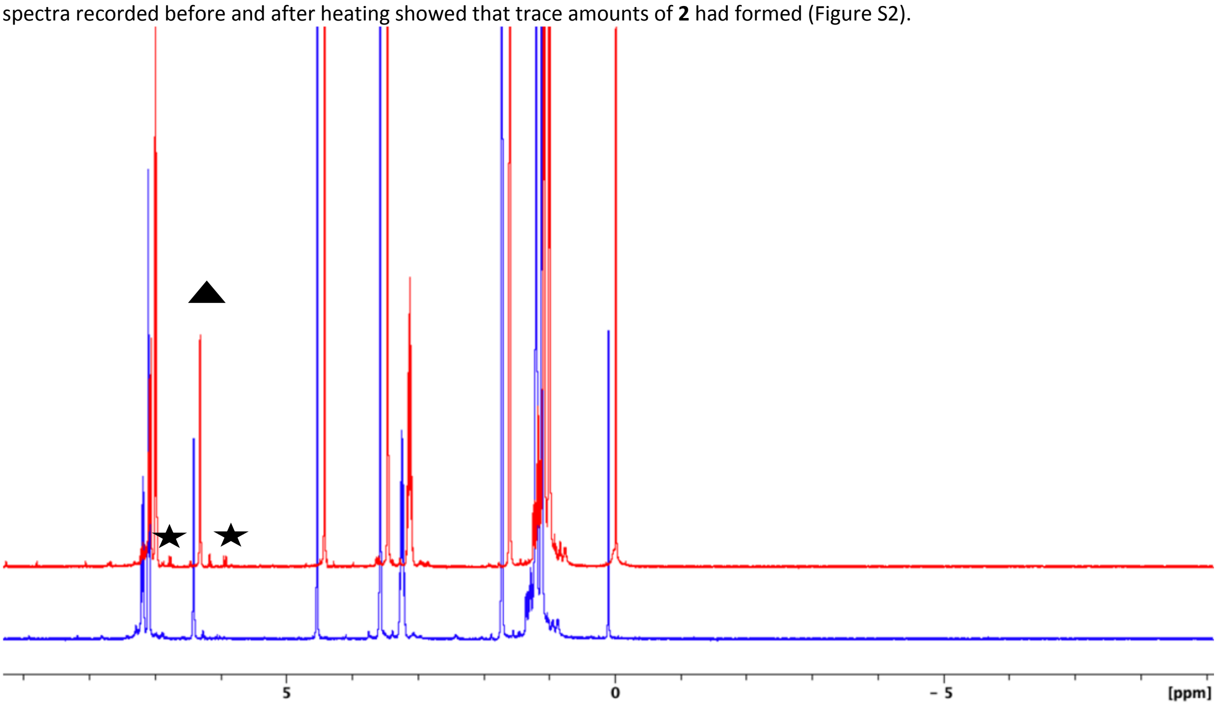

Figure S2. ^{1}H NMR spectra of a solution of **4** in C_6D_6 before (blue trace) and after (red trace) heating for 300 h at 60 °C under 8 bar of H_2. The signals attributable to the NCH signals of **4** and **2** are labelled by triangles and stars, respectively.

Photolysis of 4 under H_2, D_2, or a mixture of H_2/HD/D_2.

A solution of **4** (7 mg, 7 µmol) in THF-D_8 (0.5 mL) was transferred to a medium-walled high pressure NMR tube and degassed by completing three freeze-pump-thaw cycles. The NMR tube was then pressurized with the appropriate gas (8 bar H_2, D_2, or a mixture of all isotopomers, respectively) and subsequently irradiated for 7.5 h with a medium pressure mercury lamp. The reaction was monitored by recording NMR spectra before and after the irradiation period (Figures S3 – S9).

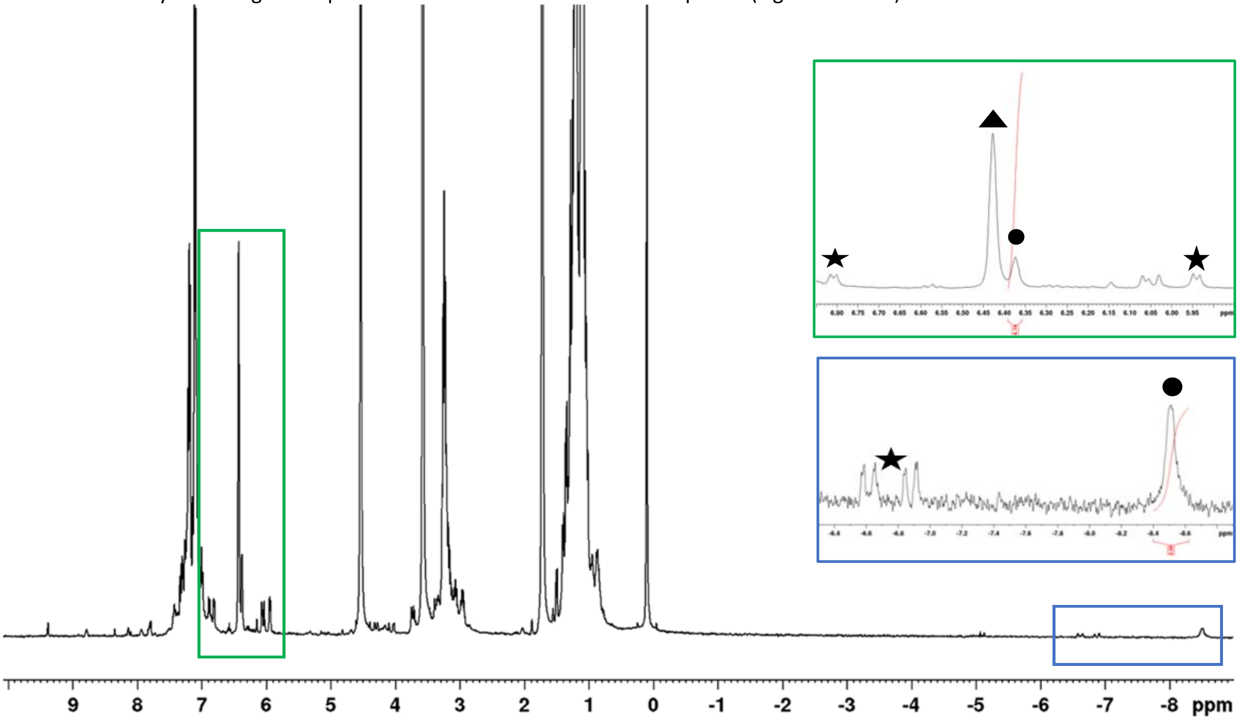

Figure S3. ^{1}H NMR spectrum of a solution of **4** in THF-D_8 after 7.5 h of irradiation with a medium pressure mercury lamp under 8 bar of H_2 with expansions showing the regions attributable to NCH and CrH resonances. Labelled signals are attributable to **4** (triangle), **2** (star) and **6** (circle), respectively.

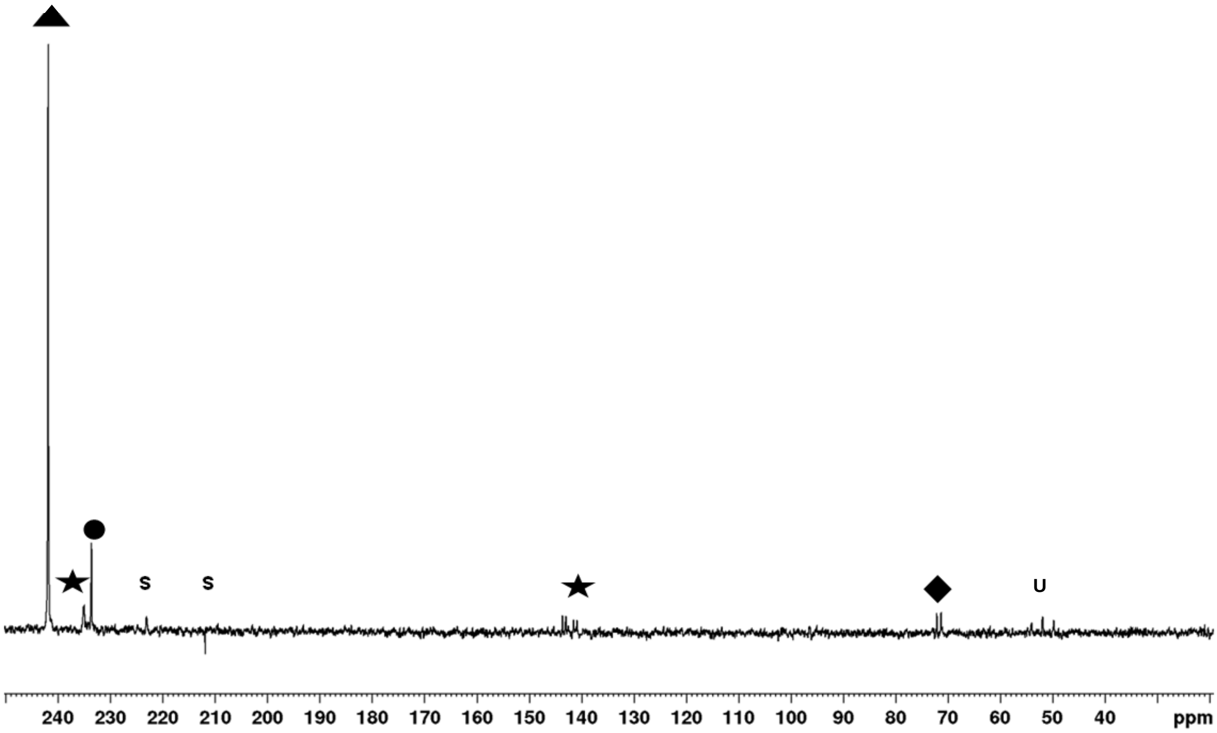

Figure S4. ^{31}P NMR spectrum of a solution of **4** in THF-D_8 after 7.5 h of irradiation with a medium pressure mercury lamp under 8 bar of H_2. Labelled signals are attributable to 4 (triangle), 2 (star), 6 (circle), and 1 (diamond) respectively. Features marked S are electronic spikes and U the signals of unknown by-products.

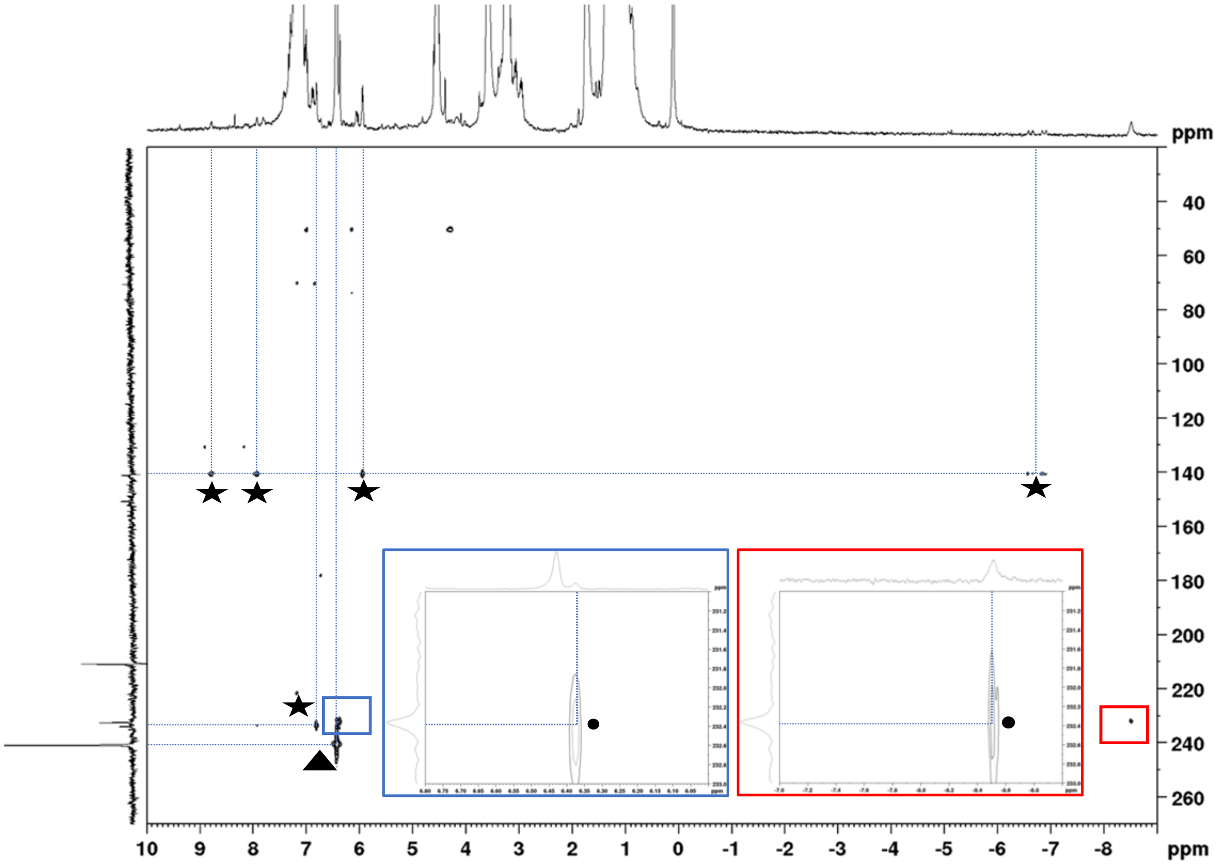

Figure S5. $^{1}H,^{31}P$-HMQC NMR spectrum and expansions of a solution of **4** in THF-D_8 after 7.5 h of irradiation with a medium pressure mercury lamp under 8 bar of H_2. Labelled signals are attributable to **4** (triangle), **2** (star), **6** (circle), respectively.

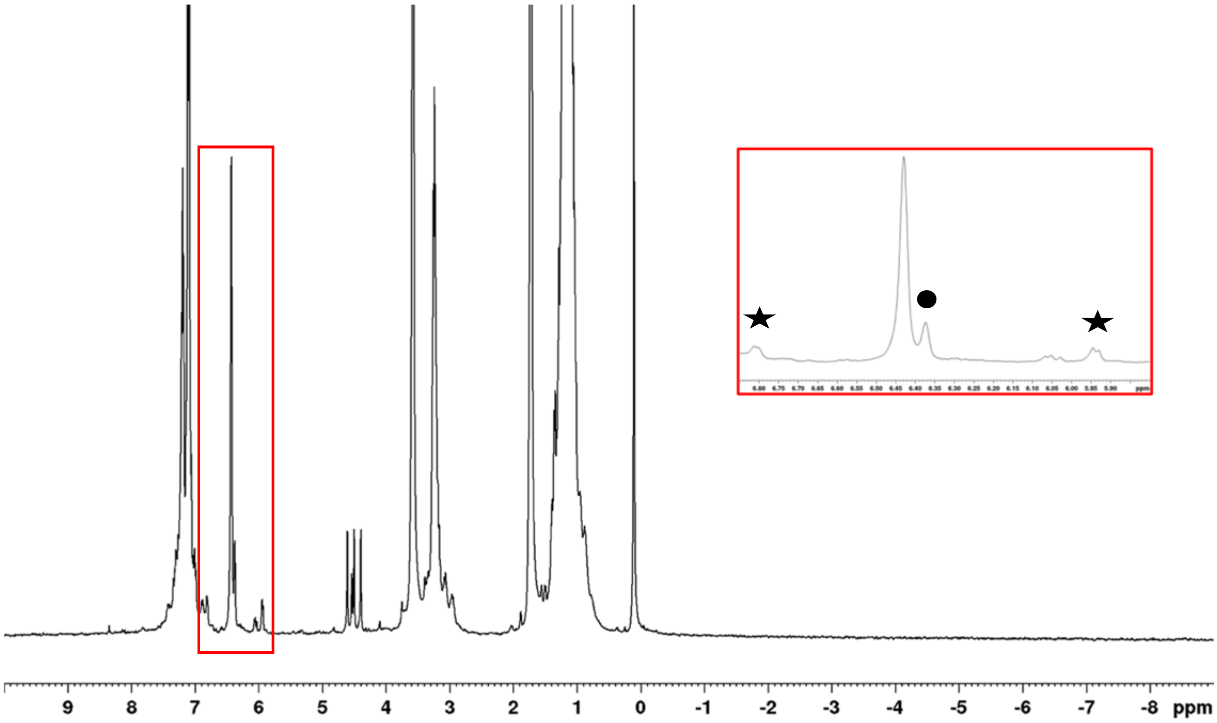

Figure S6. ^{1}H NMR spectrum of a solution of **4** in THF-D_8 after 7.5 h of irradiation with a medium pressure mercury lamp under 8 bar of D_2. Labelled signals are attributable to **2** (star) and **6** (circle), respectively. The presence of the NCH and simultaneous absence of the hydride resonances of **2** and **6** proves the incorporation of D_2 from the gas phase.

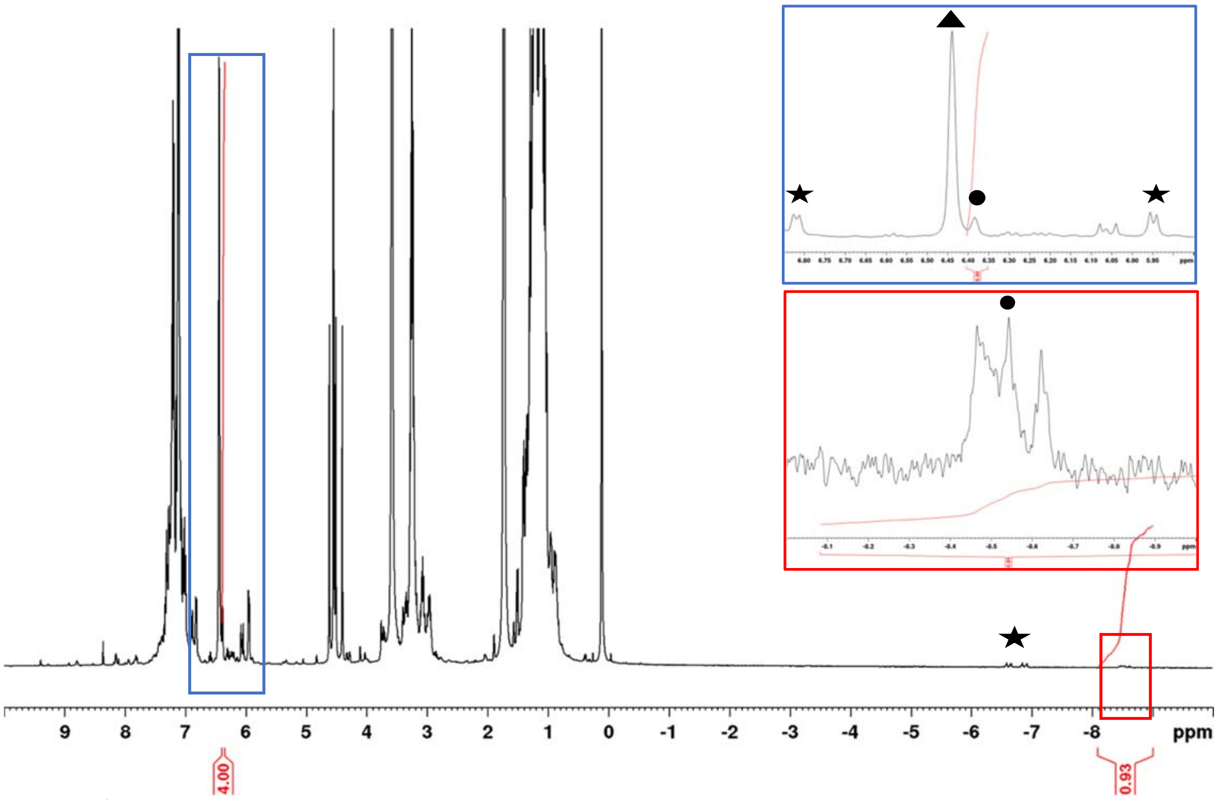

Figure S7. ^{1}H NMR spectrum of a solution of **4** in THF-D_8 after 7.5 h of irradiation with a medium pressure mercury lamp under 8 bar of a mixture of dihydrogen isotopomers. Labelled signals are attributable to **2** (star) and **6** (circle), respectively.

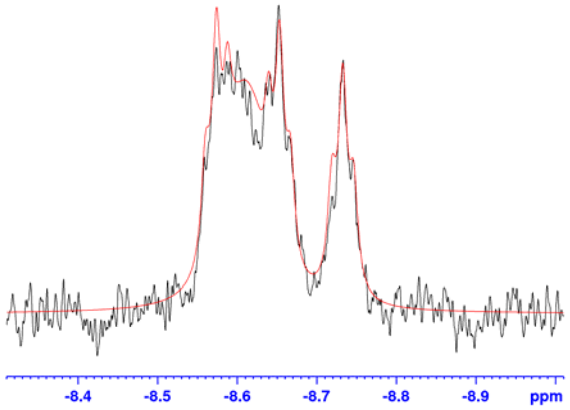

Figure S8. Hydride region of the ^{1}H NMR spectrum of a solution of **4** in THF-D_8 after 7.5 h of irradiation with a medium pressure Hg-lamp under 8 bar of a mixture of dihydrogen isotopomers (blue trace) and simulation (red trace) as a superposition of signals attributable to **6** and **6**-D_1 (ratio 0.56:1) with J_{HD} = 31.8 Hz and J_{HP} = 5.5 Hz. The increased linewidth of the signal of **6** ($\Delta\nu_{1/2}$ 18.6 vs. 4.8 Hz) reflects an additional relaxation contribution arising from dipolar coupling between the two ^{1}H nuclear spins in **6**, which is absent in the D_1-isotopomer.

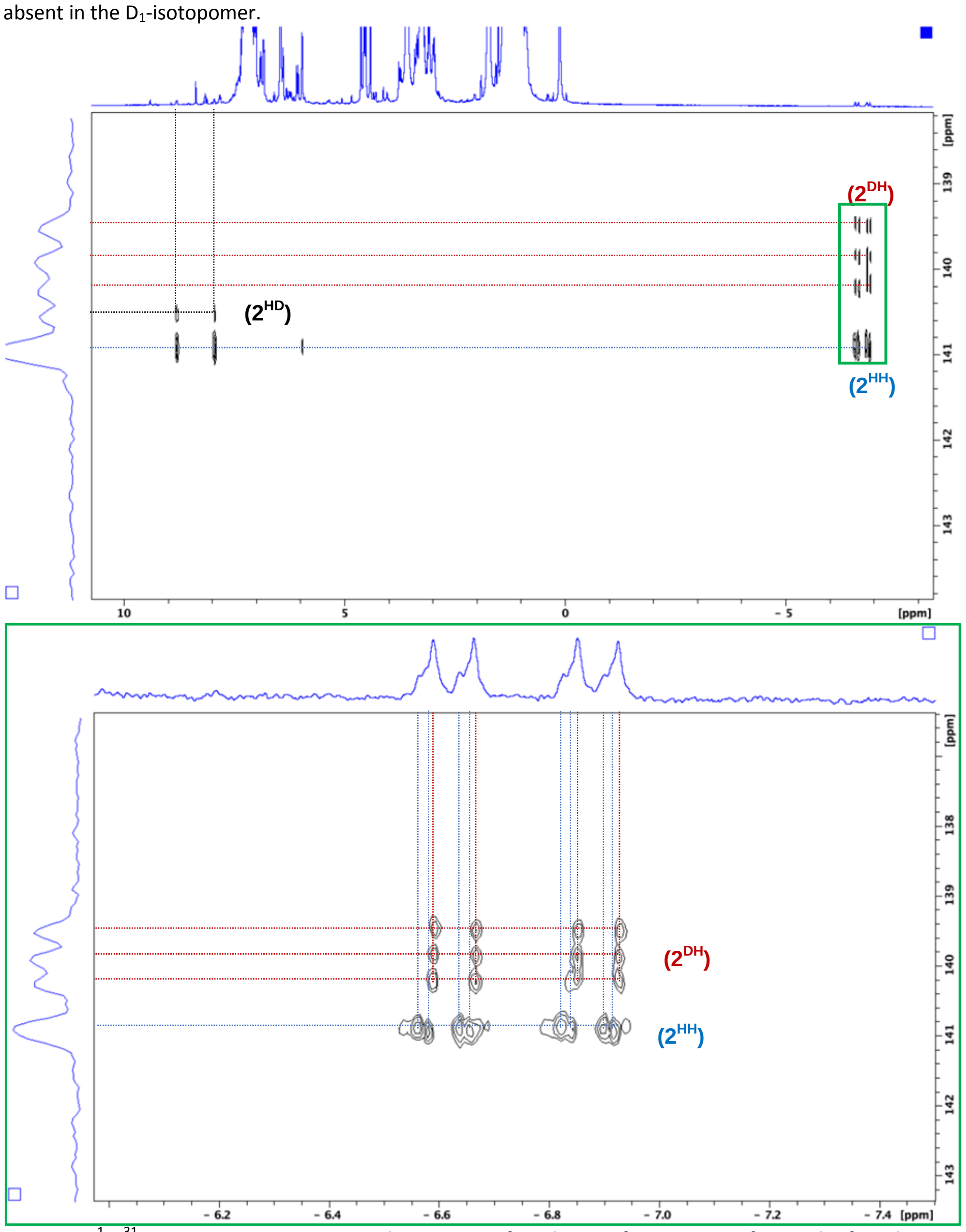

Figure S9. ^{1}H,^{31}P-HMQC NMR spectrum and expansion of a solution of **4** in THF-D_8 after 7.5 h of irradiation with a medium pressure mercury lamp under 8 bar of a mixture of hydrogen isotopomers. Spectral labels indicate cross-peaks attributable to different isotopomers of **2**.

Kinetic study of the photolysis of 2 and 4 under H_2-pressure

A solution of **2** or **4** (7 mg, 7 µmol) in THF-D_8 (0.5 mL) was transferred to a medium-walled high pressure NMR tube and degassed by completing three freeze-pump-thaw cycles. The NMR tube was pressurized with H_2, followed by acquisition of an initial ^{1}H NMR spectrum. The solution was then irradiated for different intervals with a medium-pressure mercury lamp (up to a total irradiation time of 270 min), and a ^{1}H NMR spectrum recorded after each interval. Evaluation of the product distribution at each point in time was performed by spectral integration of suitable signals using the residual signal of the deuterated solvent as reference. Results obtained under different H_2-pressures are summarized in Tables S1 to S4. Evaluation of the data revealed that the consumption of starting material **2** during the initial reaction stages follows a pseudo-first-order rate law. The rate constants decrease with increasing H_2-pressure without that a quantitative relation becomes apparent (Figure S10). Further evaluation of the data was hampered, among others, by the appearance of an unidentified product arising most likely from decomposition.

Table S1. Product distribution over time during photolysis of **2** under 1 bar of Argon.

Irradn. [min]	**2** [a]	**4** [a]	**6** [a]	unidentified [a]
0	100	0	0	0
7	94.8	5.2	0	0
17	81.9	13.4	1.4	4.7
50	46.5	32.8	0	20.8
120	17.3	53.2	0	29.5
180	6.9	62.0	2.9	28.2
270	0	70.7	4.4	24.9

[a] normalized in % of the total integral.

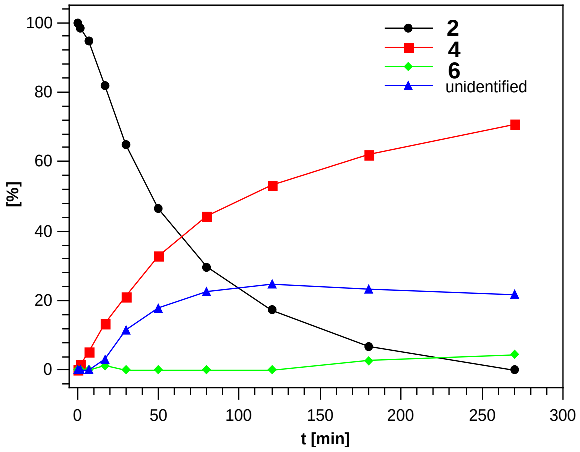

Table S2. Product distribution over time during photolysis of **2** under 3 bar of H_2.

Irradn. [min]	**2** [a]	**4** [a]	**6** [a]	unidentified [a]
0	100	0	0	0
7	94.0	2	4	0
17	87.6	4.5	7.9	0
50	60.8	11.9	16.0	11.3
120	26.1	31.6	12.8	29.5
180	17.8	42.9	11.0	28.3
270	12.1	50.4	10.2	27.3

[a] normalized in % of the total integral.

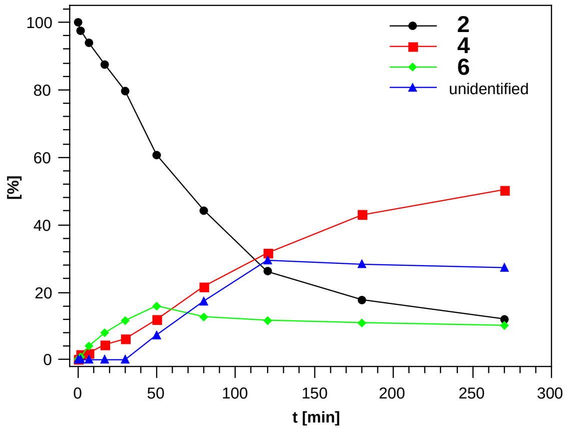

Table S3. Product distribution over time during photolysis of **2** under 8 bar of H_2.

Irradn. [min]	**2** [a]	**4** [a]	**6** [a]	unidentified [a]
0	100	0	0	0
7	92.6	2.7	4.7	0
17	86.6	4.3	9.0	0
50	65.3	14.8	20.0	5.3
120	34.1	33.9	24.9	7.1
180	21.4	42.1	24.9	10.6
270	16.4	49.9	23.7	10.1

[a] normalized in % of the total integral.

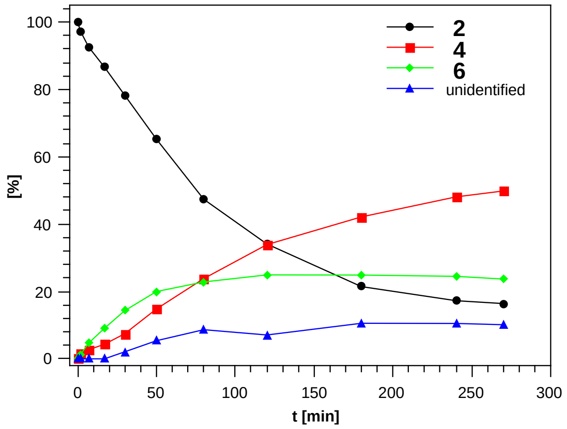

Table S4. Product distribution over time during photolysis of **4** under 8 bar of H_2.

Irradn. [min]	**4**[a]	**2**[a]	**6** [a]
0	100	0	0
7	97.5	0	2.5
17	94.2	0	5.8
50	84.9	4.2	10.9
120	73.9	10.6	15.5
180	70.5	12.8	16.7
270	66.8	13.6	19.6

[a] normalized in % of the total integral.

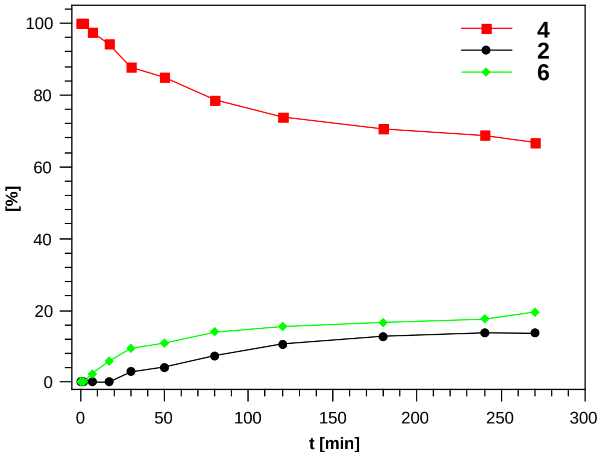

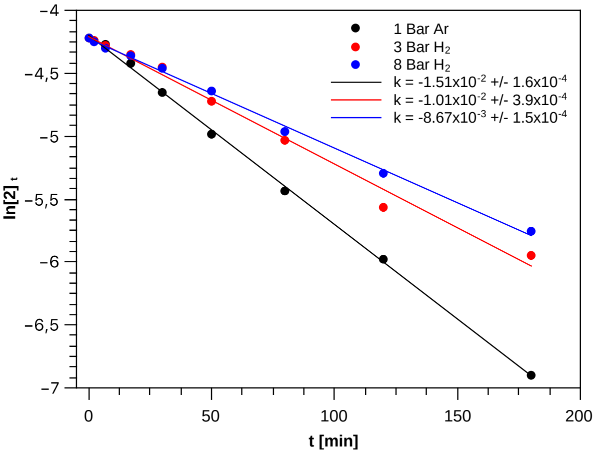

Figure S10: Plot of ln([**2**]) vs. t showing the time evolution of the concentration of **2** during the initial stages of photolysis experiments under Ar, 3 bar of H_2, and 8 bar of H_2 initial pressure. The straight lines represent the results of linear fits to the data.

Reaction of 6 with D_2

A solution of **6**-H_2 was generated photochemically from **4** and H_2 (8 bar) in a high pressure NMR tube as described above. The solution was then frozen in liq. N_2 and the NMR tube evacuated to remove gaseous H_2 and, after the solution had been allowed to warm up to ambient temperature, pressurized with D_2 (8 bar). The sample was agitated to ensure dissolution of D_2 in the liquid phase. A ^{1}H NMR spectrum run immediately afterwards showed that the NCH signals of **2** and **6** as well as the hydride signal of **2** were still visible whereas the hydride signal of **6** had disappeared, pointing out that **6** had undergone rapid H_2/D_2-exchange whereas **2** was obviously inert (Figure S11).

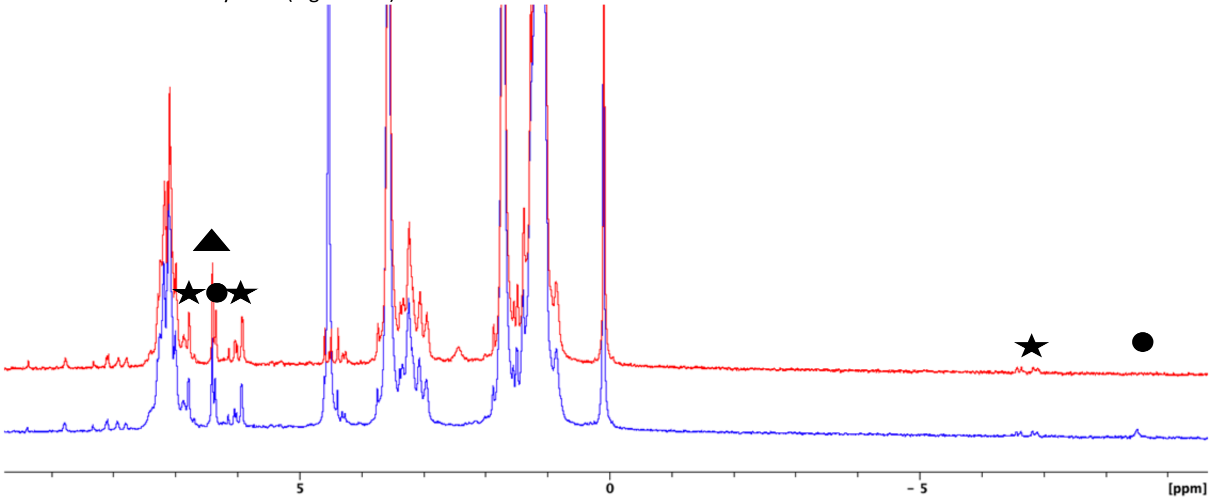

Figure S11. ^{1}H NMR spectra of a solution of **4** in THF-D_8 subjected to photolysis under 8 atm of H_2 (blue trace) and after exposure with 8 atm of D_2 (red trace). The labelled NCH and hydride resonances are attributable to **2** (star), **4** (triangle), and **6** (circle).

Thermal conversion of 6 under H_2 atmosphere

Photolysis of **4** under H_2 (8 bar) in a high pressure NMR tube was carried out as described above. A ^{1}H NMR spectrum was recorded and the sample then kept at 40 °C for 336 h without further irradiation, with small interruptions in regular intervals to acquire NMR spectra for monitoring the progress of the reaction. Analysis of the composition of the mixture at each point in time was performed by evaluating the integrals of the signals of NCH-units, using the resonance of the deuterated solvent as internal reference. The results (Table S5, Figure S12) confirm that eventual conversion of **6** to form **2** and, to a minor extent, **4**, took place.

Table S5. Evolution of product distribution in a solution containing a mixture of **2**/**4**/**6** at 40 °C under 8 bar of H_2.

time [h]	**4** [a]	**2** [a]	**6** [a]
0	70.2	11.3	18.6
0.5	70.4	12.2	17.4
1.5	70.4	14.3	15.3
3.5	71.0	15.7	12.3
10.5	71.8	21.4	6.3
30.5	72.9	23.6	3.5
103.5	73.1	24.7	2.2

[a] normalized in % of the total integral.

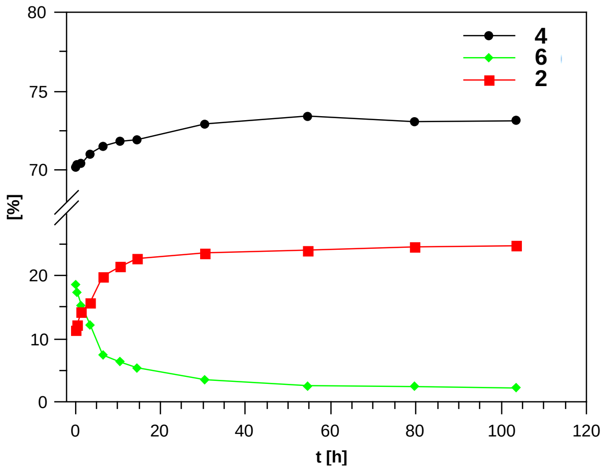

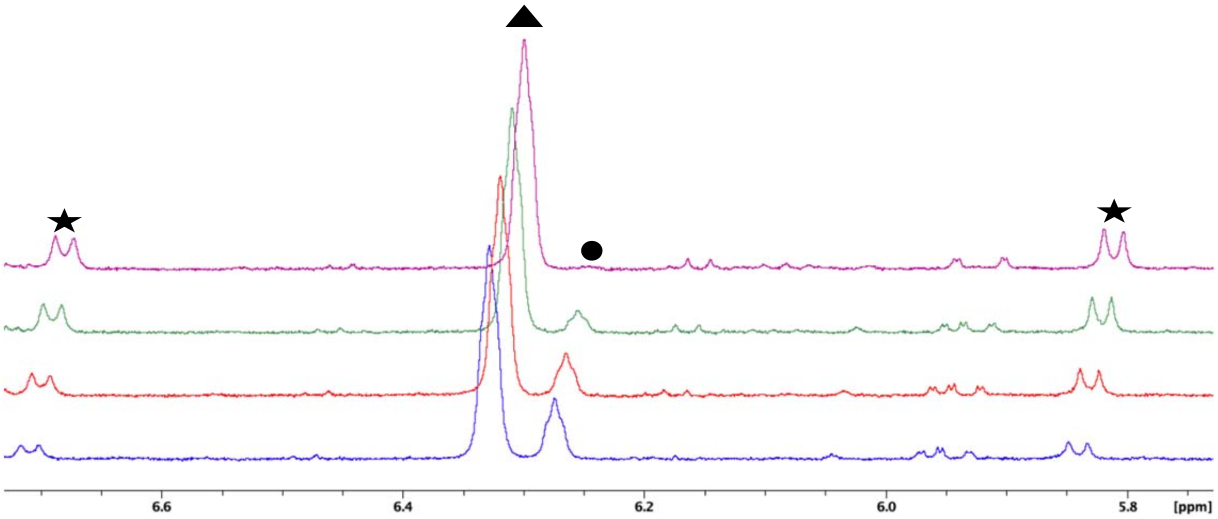

Figure S12. Expansion of ^{1}H NMR spectra of a solution of **4** in THF-D_8 under 8 bar of H_2 recorded after 2 h of photolysis (blue trace) and subsequent heating to 40 °C (without further irradiation) for 3.5 h (red trace), 10.5 h (green trace and 336 h (purple trace). Labelled signals are attributable to **2** (triangle), **4** (star), and **6** (circle), respectively.

Thermal conversion of 6 under vacuum

A solution containing **6** was generated photochemically from **4** and H_2 (8 bar) in a high pressure NMR tube as described above. A ^{1}H NMR spectrum was recorded and the sample then frozen in liq. N_2. The NMR tube was evacuated and then allowed to warm to ambient temperature, heated to 40 °C under autogenous pressure of the solvent, and kept at this temperature for 516 h without further irradiation with small interruptions to acquire NMR spectra for monitoring the progress of the reaction. The composition of the mixture was analyzed at each point in time by evaluating the integrals of the signals of NCH-units, using the resonance of the deuterated solvent as internal reference. The results (Table S6, Figure S13) confirm the eventual conversion **6**→ **4**.

Table S6. Evolution of product distribution in a solution containing a mixture of **2**/**4**/**6** at 40 °C in the absence of gaseous H_2.

Reaction time [h]	**4** [a]	**2** [a]	**6** [a]
0	72.9	11.4	15.7
1.5	74.1	11.4	14.6
18	75.6	12.2	12.2
66	77.5	12.8	9.7
200	81.0	12.7	6.3
372	84.2	12.8	3.0
516	86.3	12.0	1.7

[a] normalized in % of the total integral.

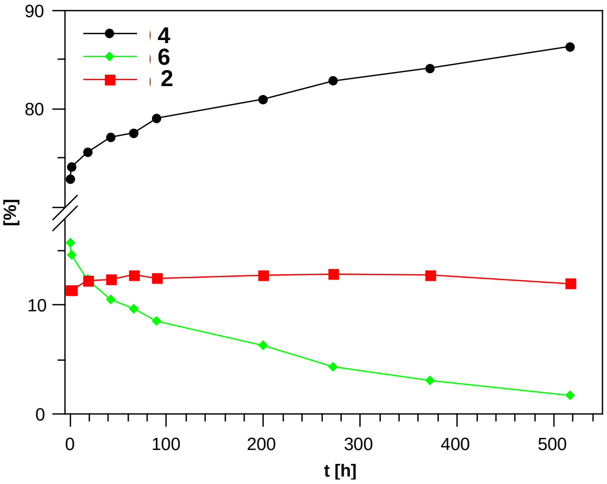

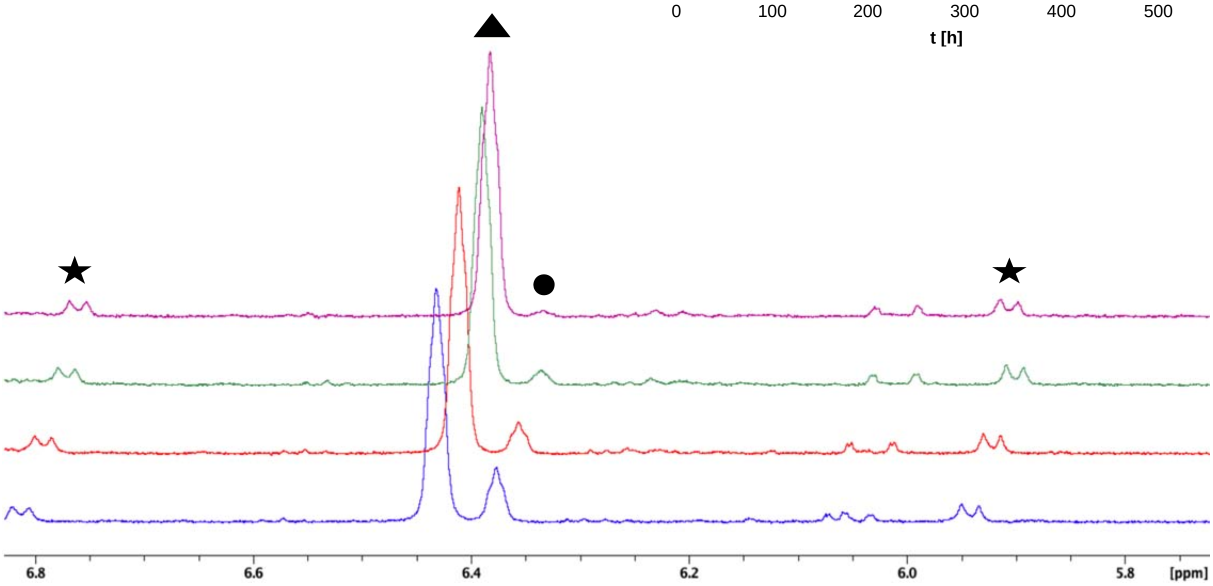

Figure S13. Expansion of ^{1}H NMR spectra of a solution of **4** in THF-D_8 recorded after 2 h of photolysis under 8 bar of H_2 and removal of gaseous H_2 (blue trace) and subsequent heating to 40 °C (without further irradiation) for 90 h (red trace), 272 h (green trace and 516 h (purple trace). Labelled signals are attributable to **2** (triangle), **4** (star), and **6** (circle), respectively.

Hydrogenation of Styrene

(a) Styrene (1.5 µl, 1.4 mg, 13 µmol) was added to a solution of **4** (2 mg, 2 µmol, 16 mol-% based on styrene) in THF-D_8 (0.5 mL). The sample was transferred to a medium-walled high pressure NMR tube and degassed by completing with three freeze-pump-thaw cycles. The NMR tube was then pressurized with H_2 (8 bar initial pressure). After acquisition of a reference ^{1}H NMR spectrum, the solution was irradiated for 11.5 h with a medium pressure Hg lamp, with short interruptions to acquire ^{1}H NMR spectra for reaction monitoring. Analysis of the composition of the mixture at each point in time was performed by evaluating the integrals of the signals of NCH-units and the CH_2-signal of ethylbenzene, using a silicone resonance as internal reference (see Table S7). Near quantitative conversion of styrene to ethylbenzene (95% based on the original amount of styrene) occurred within 11.5 h of irradiation time (Figure S14). Analysis of the phosphorus-containing species at the end of the reaction revealed the presence of **4** (45% based on the amount originally present), **6** (10%), secondary diazaphospholene **1** (24%) and a species **X** (21%) tentatively identified as P-phenethyl-substituted diazaphospholene arising from phosphination of styrene by **1** (Table S7, Figure S15, S18).

(b) Hydrogenation of styrene (3 µL, 2.7 mg, 26 µmol) in THF-D_8 (0.5 mL) in the presence of **4** (1 mg, 1 µmol, 4 mol-% based on styrene) was carried out as described above to result in 71% conversion (Figure S16). Analysis of the phosphorus-containing species at the end of the reaction indicated that only minor amounts of phosphenium complexes (8% of **4** and traces of **6**) were

still present and that extensive deactivation to give **1** (30%) and **X** (62%) was observable (Table S8, Figure S17).

(c) To try identifying the catalytically active species in the hydrogenation, a sample containing styrene and **4** (16 mol-%) under H_2 (8 bar) was prepared as described above and irradiated for 20 min with a medium-pressure Hg lamp. An NMR-spectroscopic analysis confirmed that a significant amount of **6** (7%) and a small amount of ethylbenzene (16%; both values based on the amount of **4** initially present, see Figure S19) had formed. The solution was then stored at 20 °C for 18 h without further irradiation. Subsequent NMR spectroscopic analysis revealed that **4** had been completely consumed while the amount of ethylbenzene had increased (Figure S19). Signals of **1** and **X** were not detectable, suggesting negligible decay of the NHP complex during the rather short photolysis time. Quantitative evaluation of the changes in signal integrals allowed us to calculate a turnover number TON = Δn(ethylbenzene)/Δn(**6**) = 9.6 which suggests that the dihydrogen complex transfers H_2 to the substrate in a thermal reaction and can perform several turnovers without additional photochemical activation.

(d) Control experiments were carried out using the same procedure as described above. Employing $Cr(CO)_3$(naphthalene) (2 mg, 7.6 µmol, 16 mol-% based on styrene) as pre-catalyst resulted in 18 % conversion of styrene to ethylbenzene after 17 h of irradiation (Figure S20), implying an essentially stoichiometric reaction. No formation of ethylbenzene was observed in the presence of **1** (2 mg, 4.9 µmol, 16 mol-% based on styrene, Figure S21) or in the absence of any catalyst (Figure S22).

Table S7. Reaction monitoring of the photohydrogenation of styrene (16 mol-% **4**, 8 bar H_2 initial pressure)

Reaction time [h]	**4** [a]	**6** [a]	**1** [a]	**X** [a]	Ethylbenzene [a]
0	100	0	0	0	0
2.00	66.6	13.5	6	13.9	171
3.33	54.8	15.7	10.8	18.7	345
4.33	51.3	14.9	13.5	20.3	412
6.00	47.7	15.6	15.5	21.1	495
7.00	47.9	13.4	18.2	20.4	542
8.00	44.3	13.6	19.0	23.4	578
9.75	43.0	13.4	21.9	21.8	602
11.5	44.6	9.7	24.2	21.5	617

[a] normalized to $n(\mathbf{4})_{t=0}$ = 100 %.

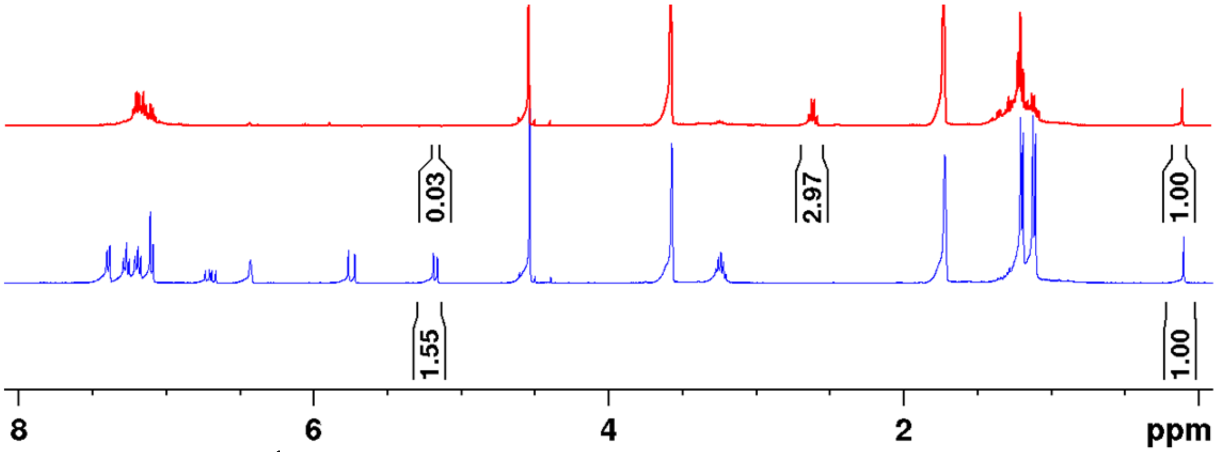

Figure S14. Expansion of the ^{1}H NMR spectrum of a solution of styrene and **4** (16 mol-%) in THF-D_8 under 8 bar of H_2 before (blue trace) and after (red trace) 11.5 h of irradiations. Integrals refer to a CH signal of styrene and the CH_2 group of ethyl benzene relative to a silicone standard.

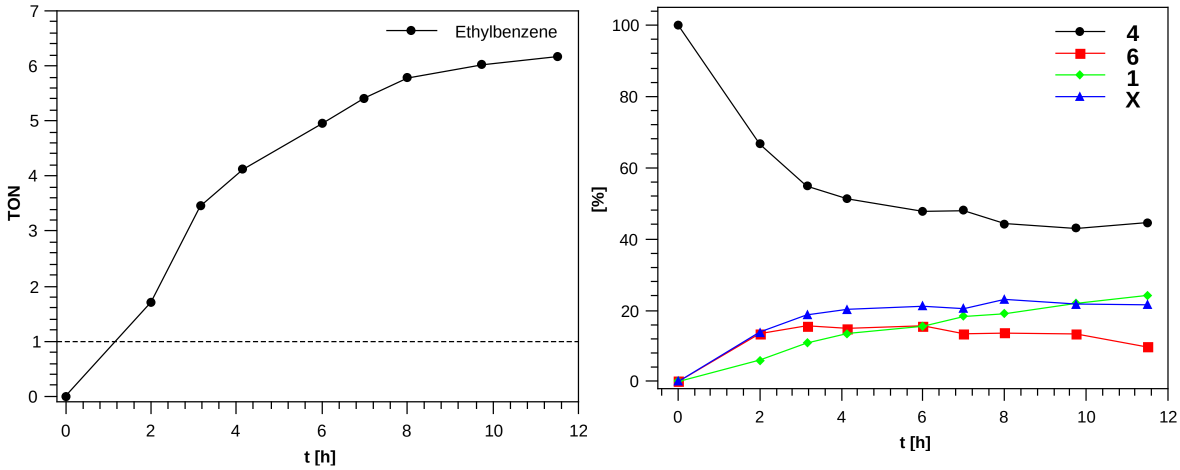

Figure S15. Kinetic plot showing the time evolution of ethylbenzene (left) and phosphorus-containing species (right) during photohydrogenation of styrene in the presence of 16 mol-% **4** (TON = n(Ethylbenzene)/$n(\mathbf{4})_{t=0}$).

Table S8. Reaction monitoring of the photohydrogenation of styrene (4 mol-% **4**, 8 bar H_2 initial pressure).

Reaction time [h]	**4** [a]	**6** [a]	**1** [a]	**X** [a]	Ethylbenzene [a]
0	100	0	0	0	0
2.00	40.1	5.7	28.6	25.6	747
3.00	34.1	4.0	28.7	33.2	971
5.00	22.0	1.7	31.4	44.9	1440
60	15.6	1.5	31.3	51.6	1530
7.00	11.0	1.1	30.6	56.7	1570
8.75	10.2	0.9	29.8	57.7	1670
10.50	8.5	0.4	29.6	61.5	1770

[a] normalized to n(**4**)$_{t=0}$ = 100 %.

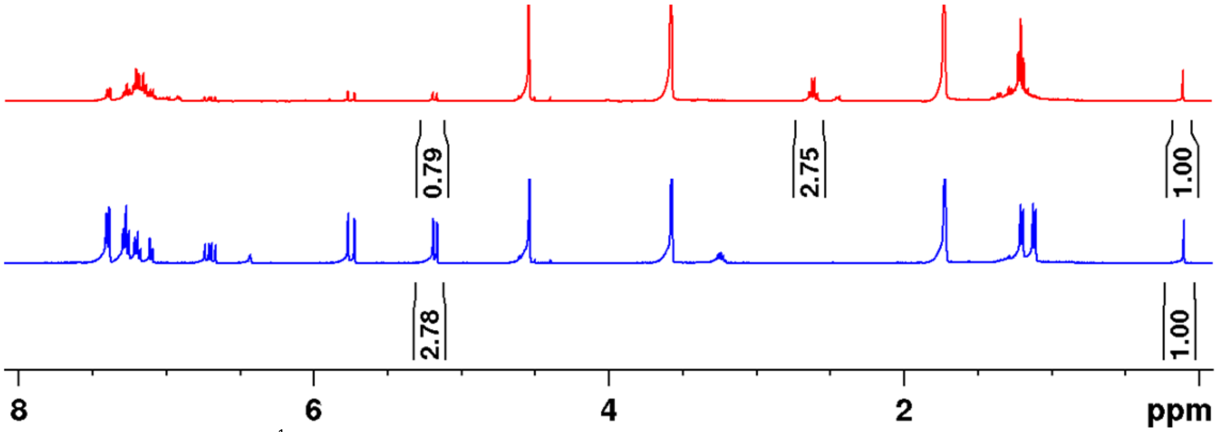

Figure S16. Expansion of the ^{1}H NMR spectrum of a solution of styrene and **4** (4 mol-%) in THF-D_8 under 8 bar of H_2 before (blue trace) and after (red trace) 10.5 h of irradiations. Integrals refer to a CH signal of styrene and the CH_2 group of ethyl benzene relative to a silicone standard.

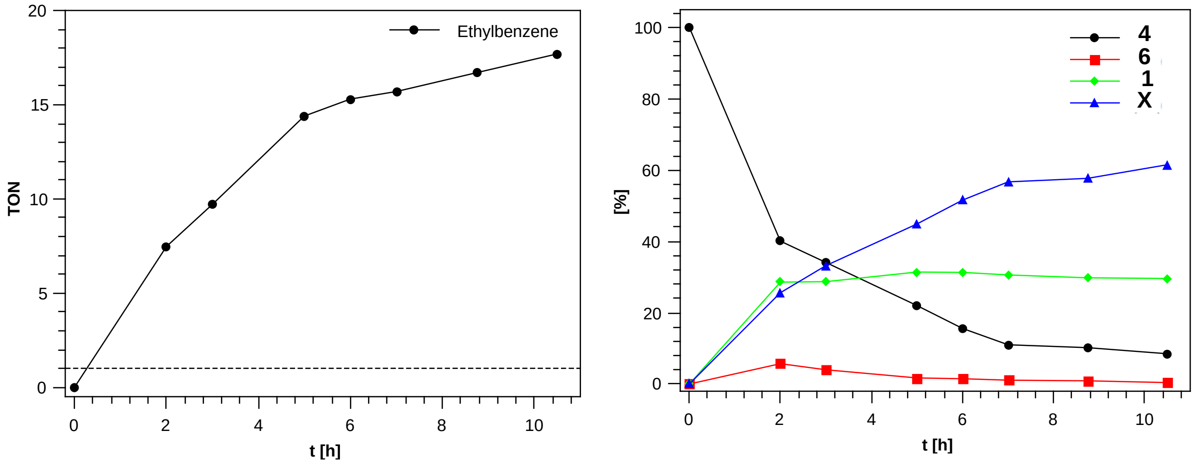

Figure S17. Kinetic plot showing the time evolution of ethylbenzene (left) and phosphorus-containing species (right) during photohydrogenation of styrene in the presence of 4 mol-% of **4** (TON = n(Ethylbenzene)/n(**4**)$_{t=0}$).

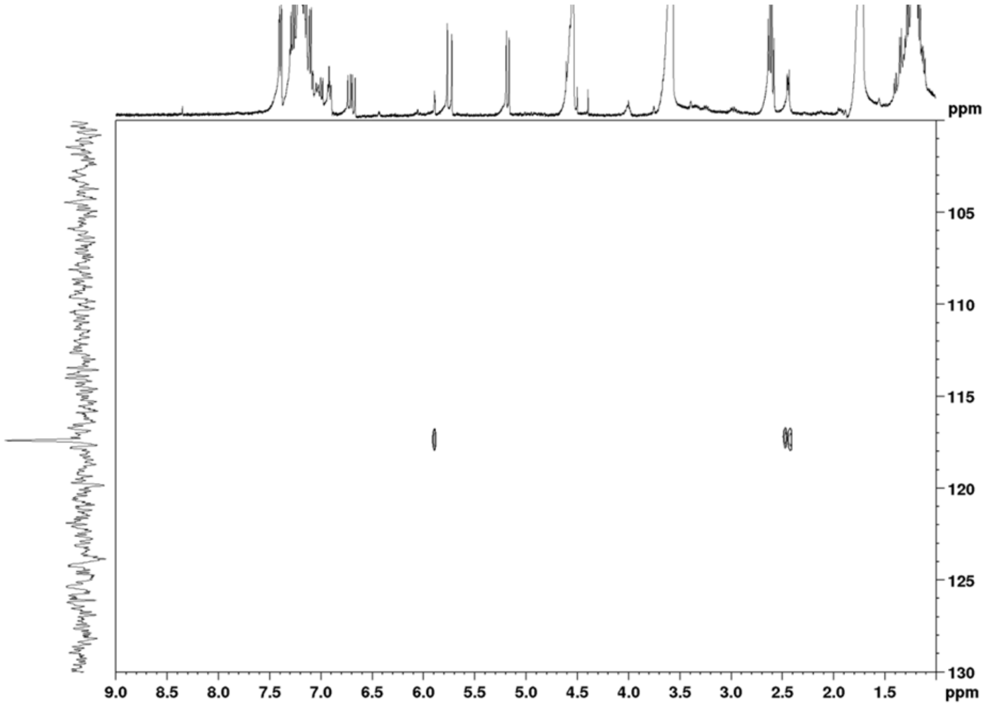

Figure S18. Expansion of the ^1H,^{31}P HMQC NMR spectrum at the end of the photo-hydrogenation of styrene in the presence of 4 mol-% of **4** showing the correlations connected with the ^{31}P NMR signal at 117 ppm attributable to **X**. The correlations result from spin coupling of the ^{31}P nucleus with protons of an NCH unit (δ^{1}H 5.8 ppm) and a $PhCH_2$-unit (δ^{1}H 2.4 ppm) and lead us to tentatively assign the molecular structure of X as a diazaphospholene bearing a P-CH_2CH_2Ph substituent.

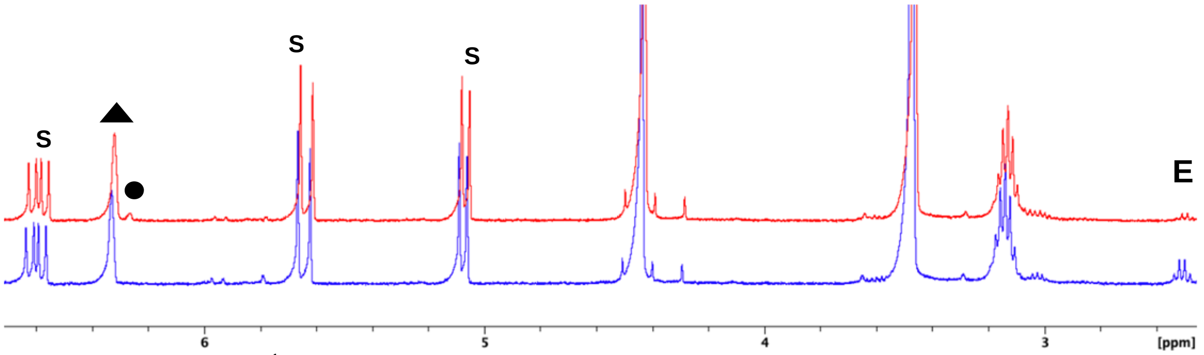

Figure S19. Expansion of the ^1H NMR spectrum of a solution of **4** and styrene in THF-D_8 after 20 min of irradiation under 8 bar of H_2 (red trace) and after storage of the solution for 18 h at 20 °C (blue trace). Labelled signals are attributable to styrene (S), ethylbenzene (E), **4** (triangle), and **6** (circle), respectively.

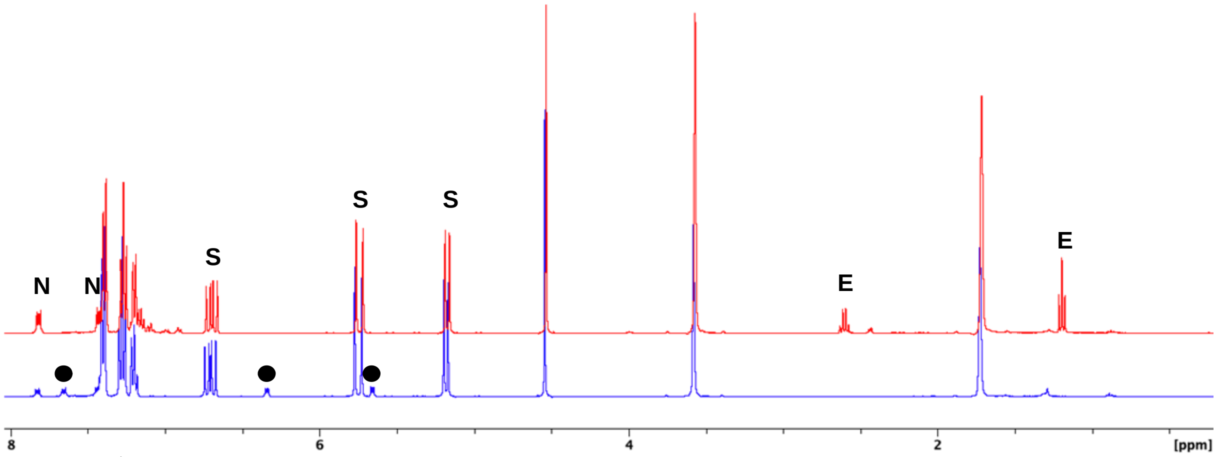

Figure S20. ^1H NMR spectra of a solution of styrene and [$Cr(CO)_3$(naphthalene)] in THF-D_8 under 8 atm of H_2 before irradiation (blue trace) and after 17h of irradiation with a medium pressure Hg lamp (red trace). Labelled signals are attributable to styrene (S), ethylbenzene (E), [$Cr(CO)_3$(naphthalene)] (circle) and naphthalene (N).

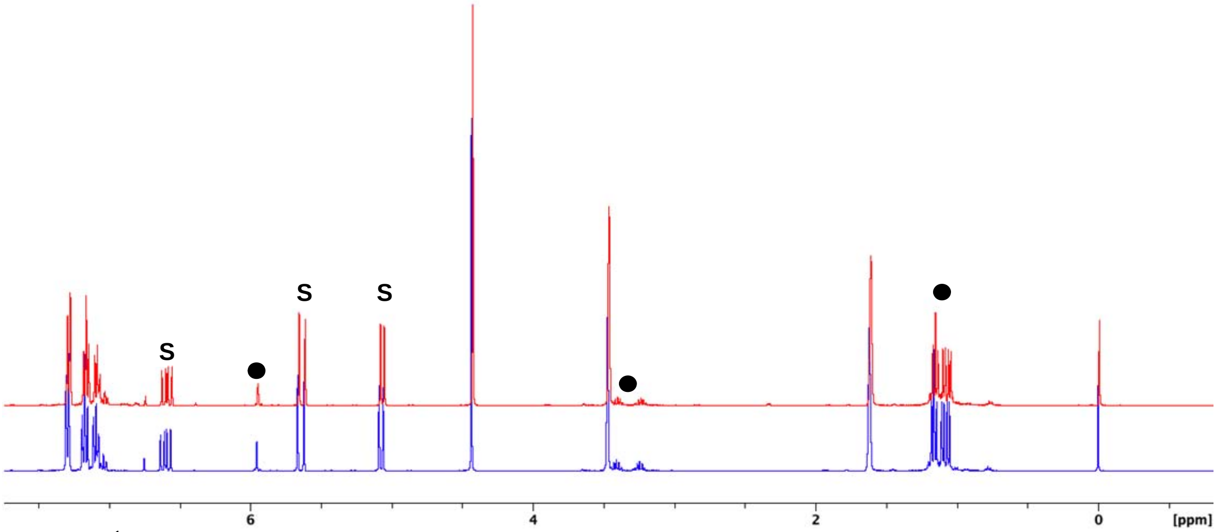

Figure S21 ^{1}H NMR spectra of a solution of styrene and **1** n THF-D$_8$ under 8 atm of H_2 before (blue trace) and after 17h of irradiation with a medium pressure Hg lamp (red trace). Labelled signals are attributable to styrene (S) and **1** (circle).

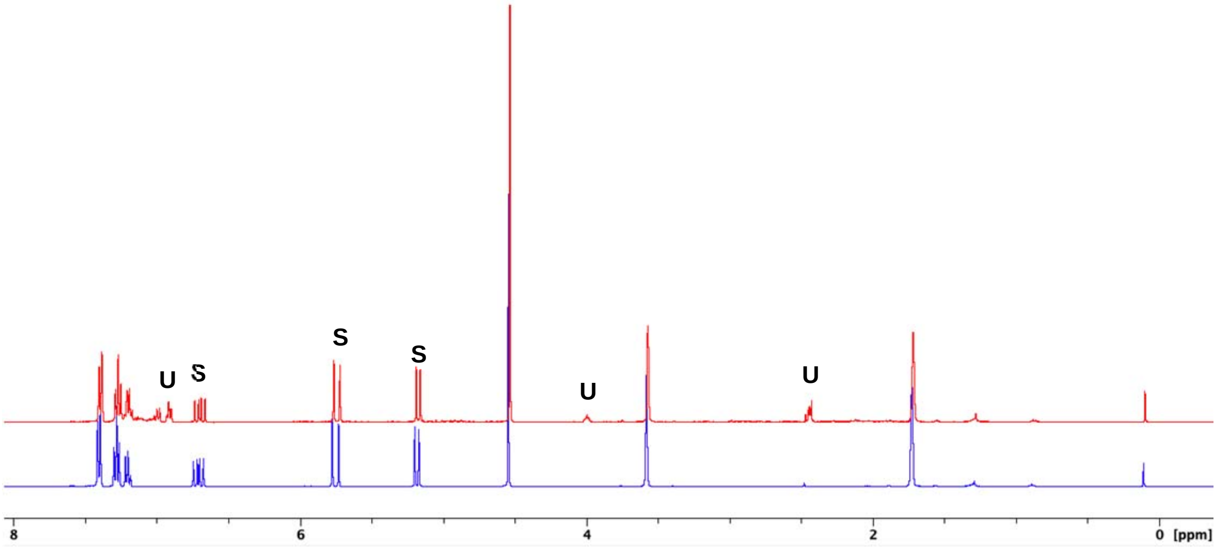

Figure S22. ^{1}H NMR spectra of a solution of styrene in THF-D$_8$ under 8 atm of H_2 before (blue trace) and after 17 h of irradiation with a medium pressure Hg lamp (red trace). Labelled signals are attributable to styrene (S) and an unidentified by-product (U).

NMR Spectra

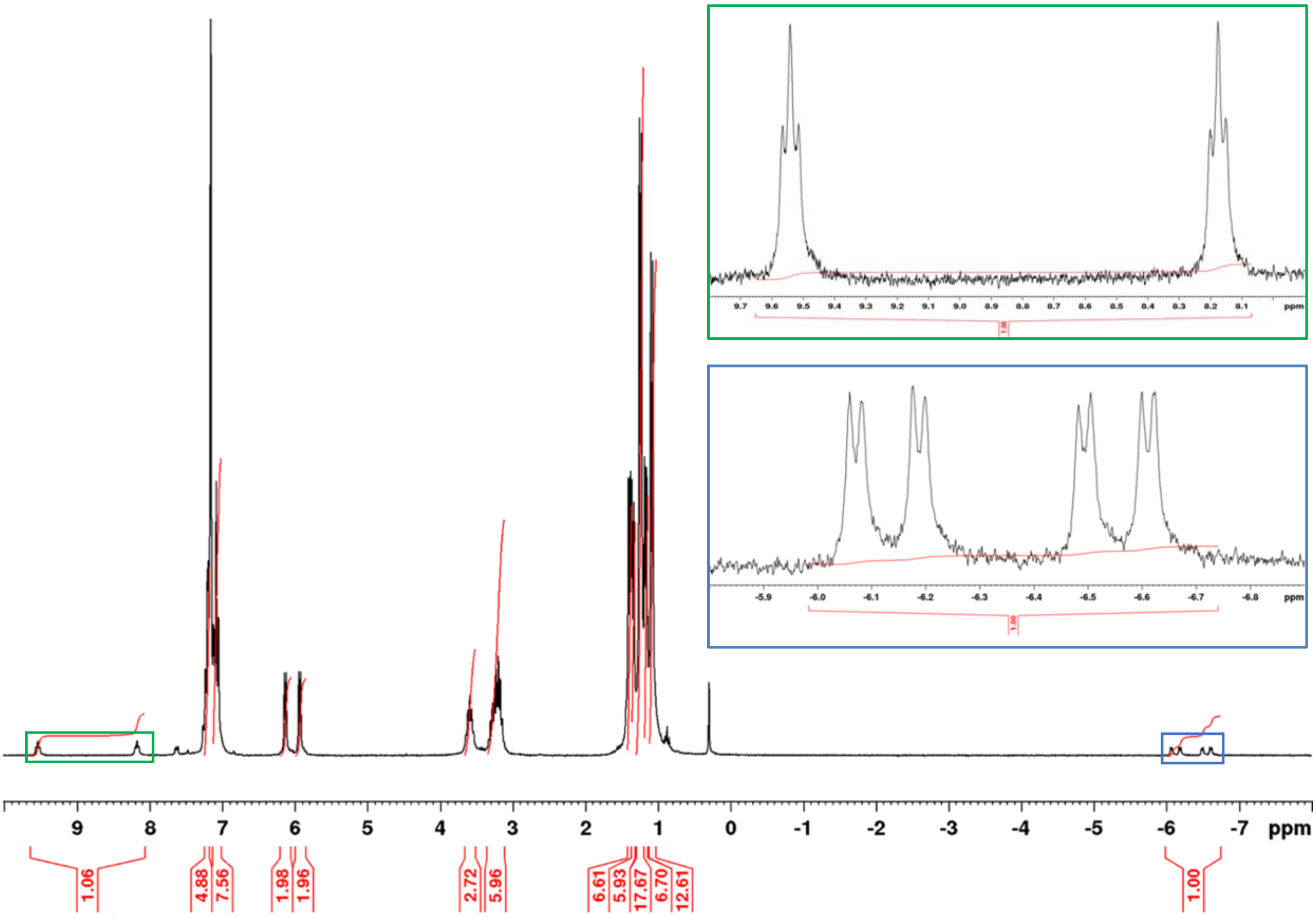

Figure S23. ^{1}H NMR spectrum of **2** in C_6D_6 with expanded regions displaying the PH and CrH signals.

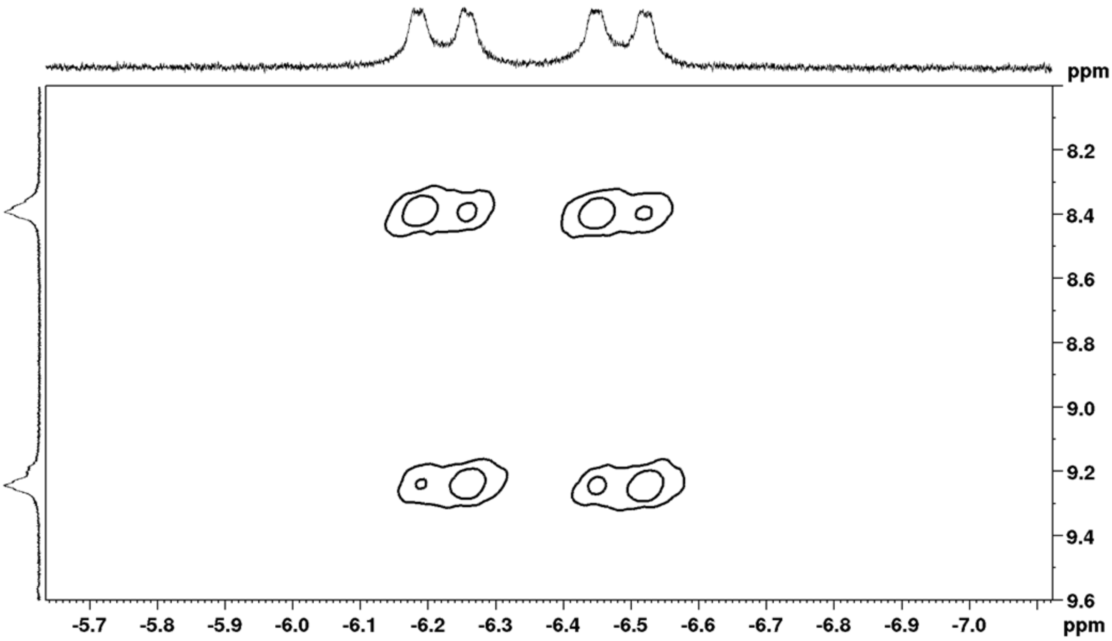

Figure S24. Expansion of the ^{1}H-EXSY NMR spectrum of **2** in C_6D_6 showing the exchange cross-peak between PH and CrH signals.

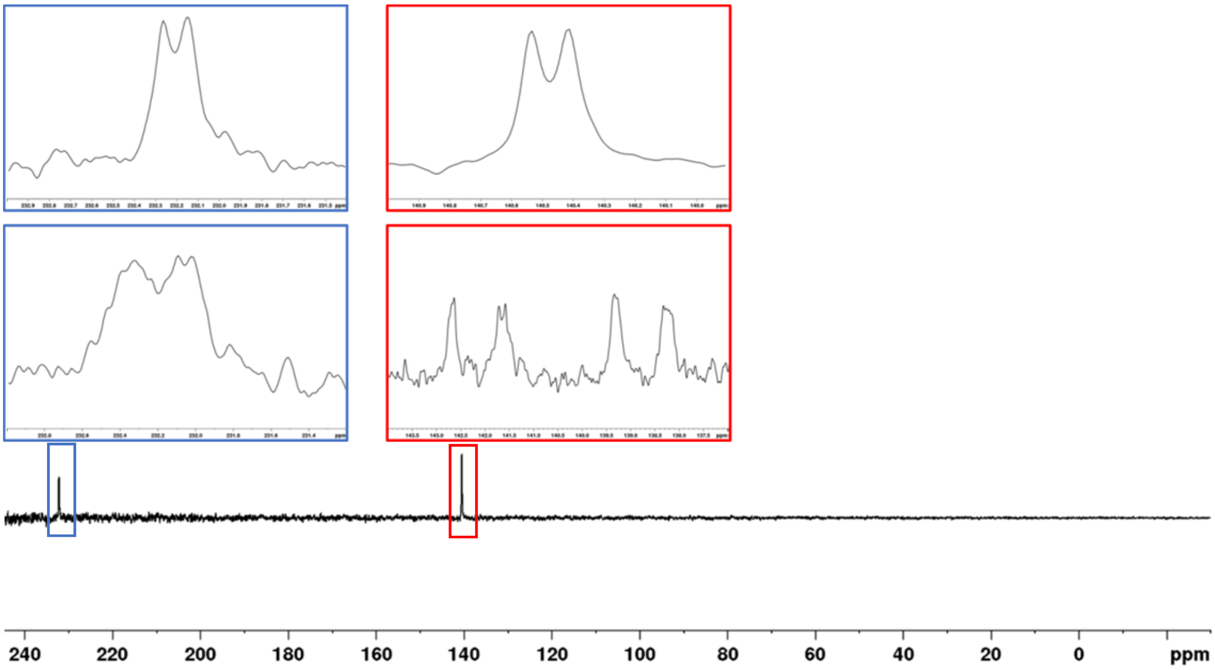

Figure S25. $^{31}P\{^1H\}$ NMR spectrum of **2** in C_6D_6 (bottom trace). Framed inserts display expansions of the two signals in the $^{31}P\{^1H\}$ (top row) and ^{31}P (bottom row) NMR spectrum.

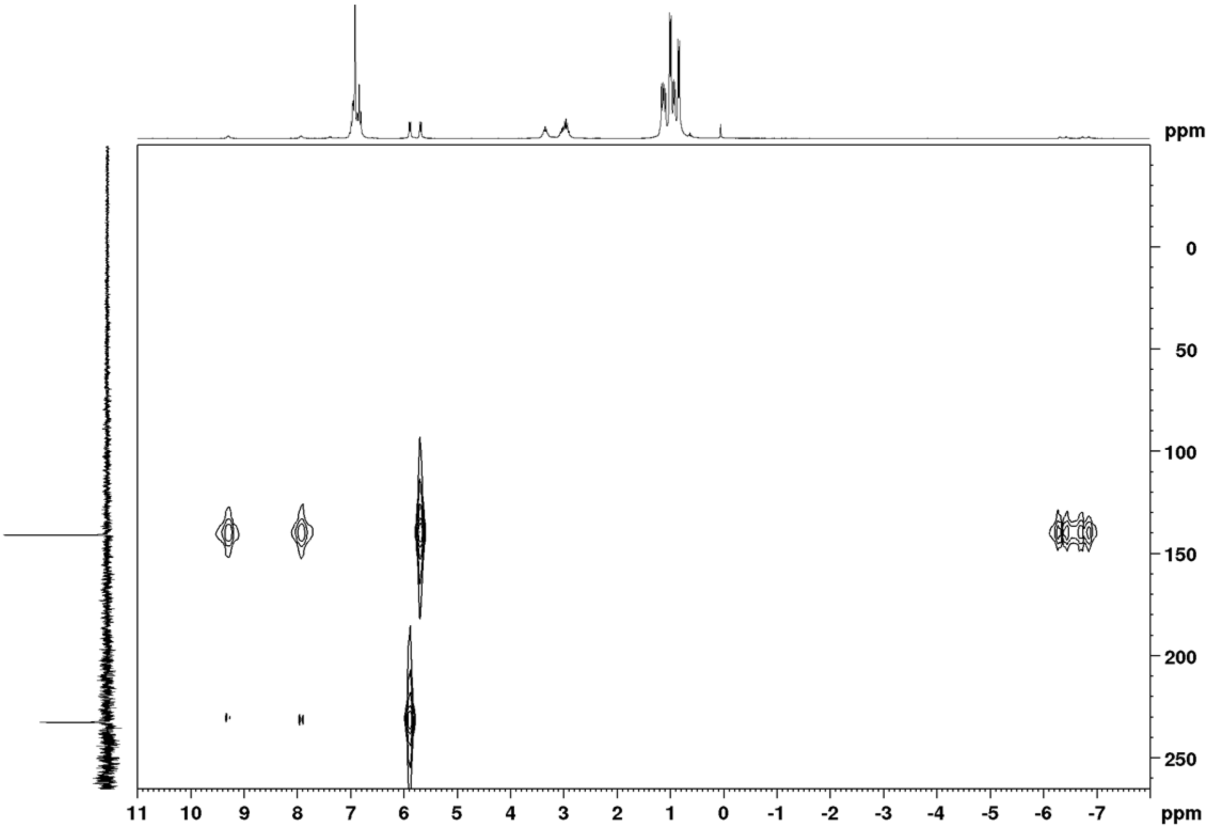

Figure S26. $^1H,^{31}P$ HMQC NMR spectrum of **2** in C_6D_6.

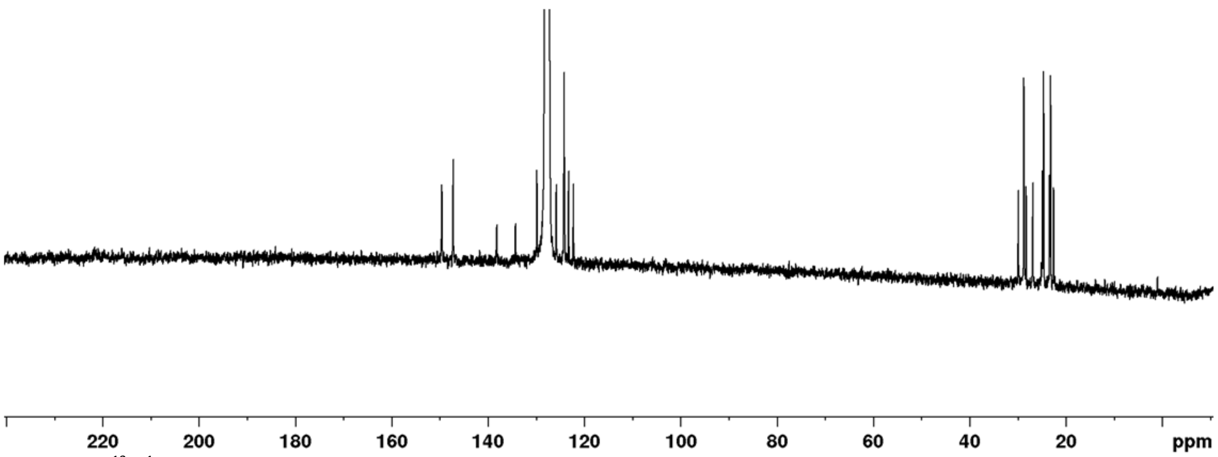

Figure S27. $^{13}C\{^1H\}$ NMR spectrum of **2** in C_6D_6.

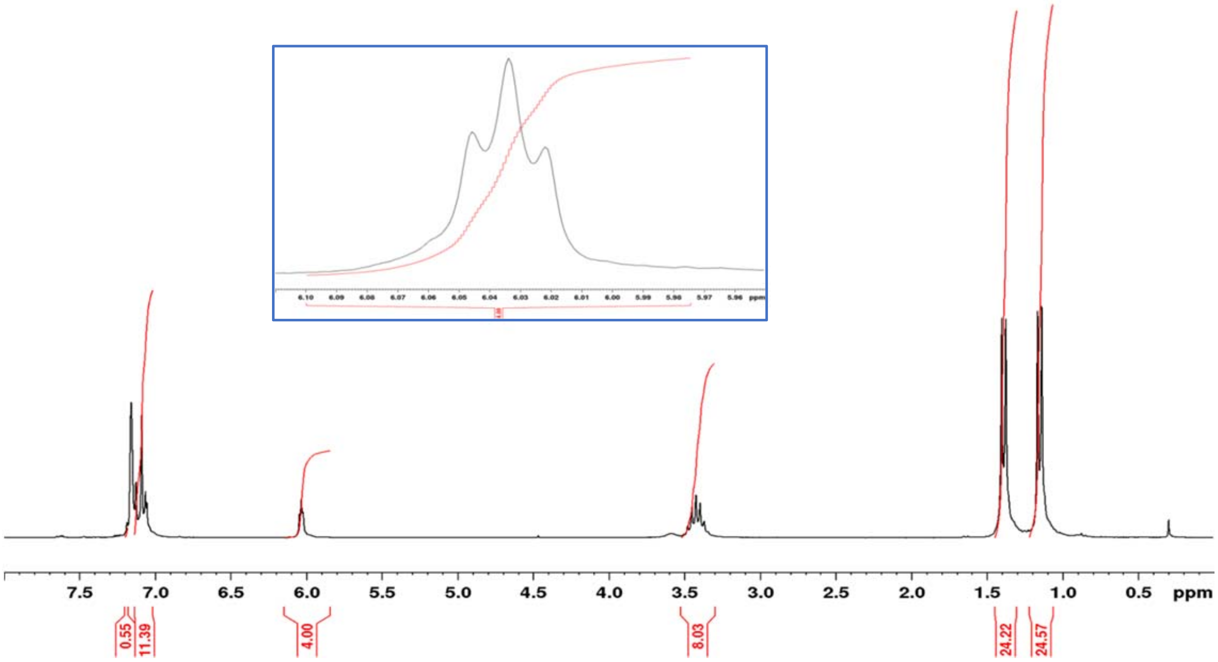

Figure S28. ^1H NMR spectrum of **4** in C_6D_6

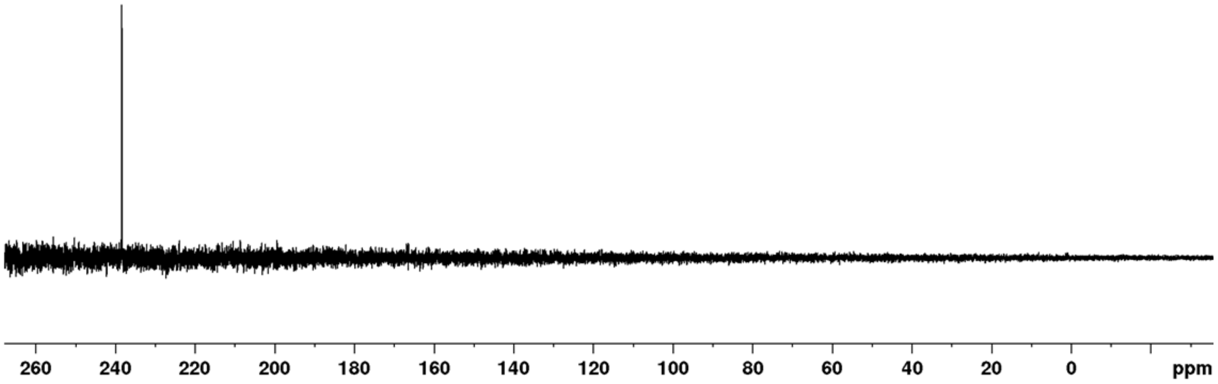

Figure S29. $^{31}P\{^1H\}$ NMR spectrum of **4** in C_6D_6

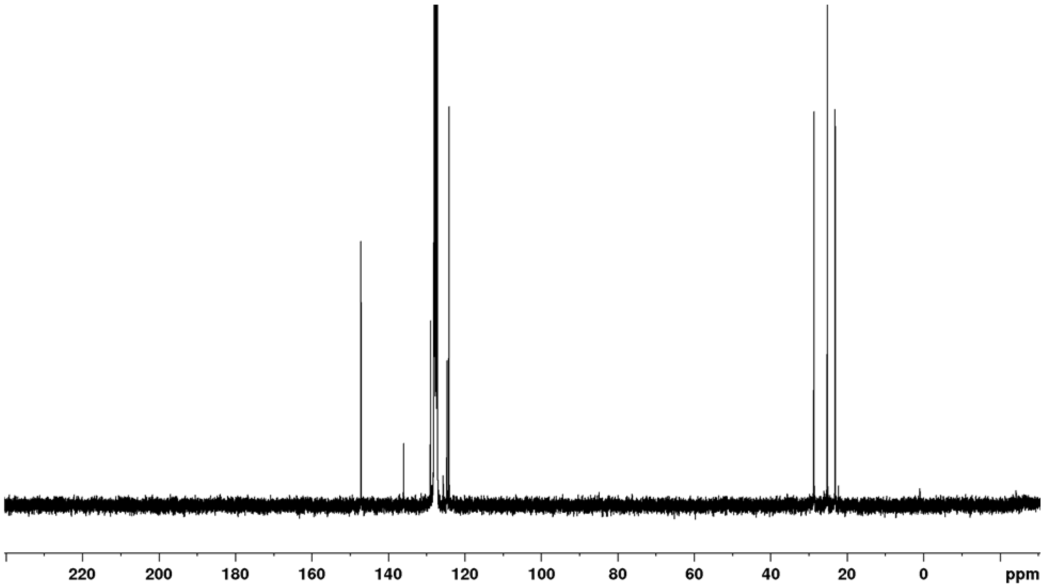

Figure S30. $^{13}C\{^{1}H\}$ NMR spectrum of **(2)** in C_6D_6

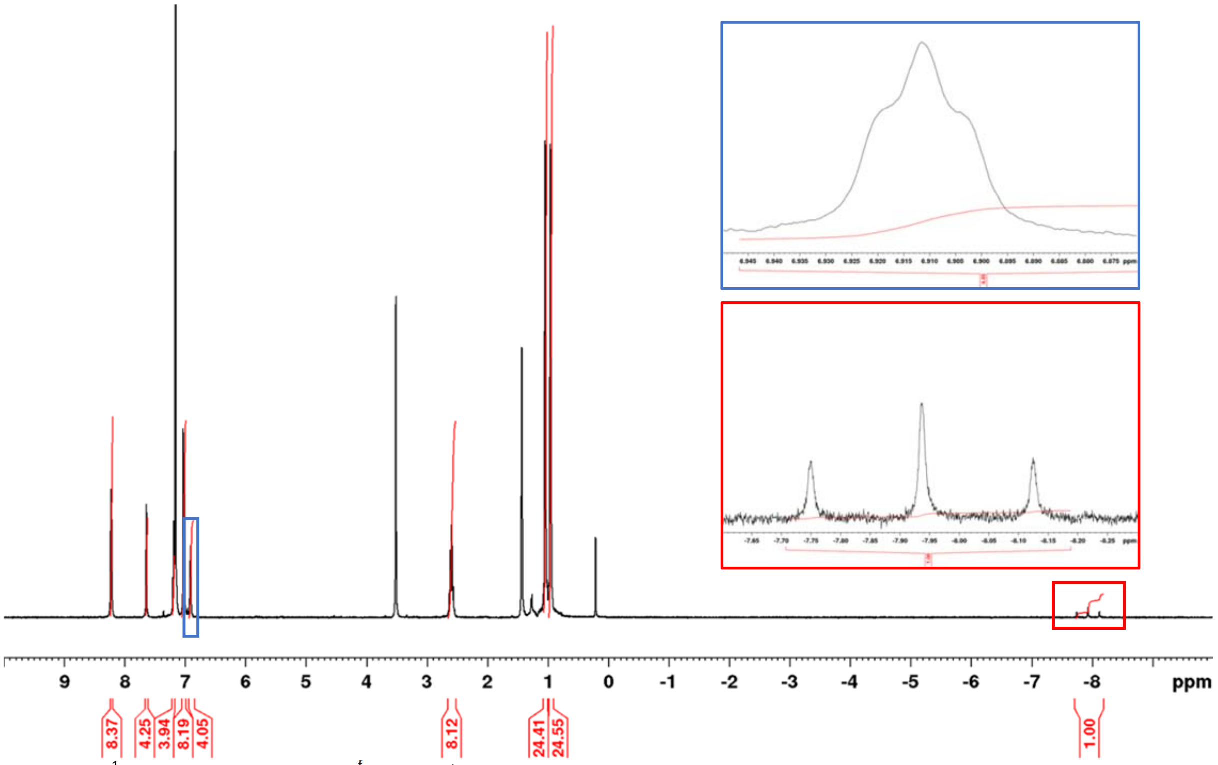

Figure S31. ^{1}H NMR spectrum of **5**[BAr$^{f}_{4}$] in C_6D_6/THF-D_8 5:1 with expansions showing the regions of the CrH and NCH signals.

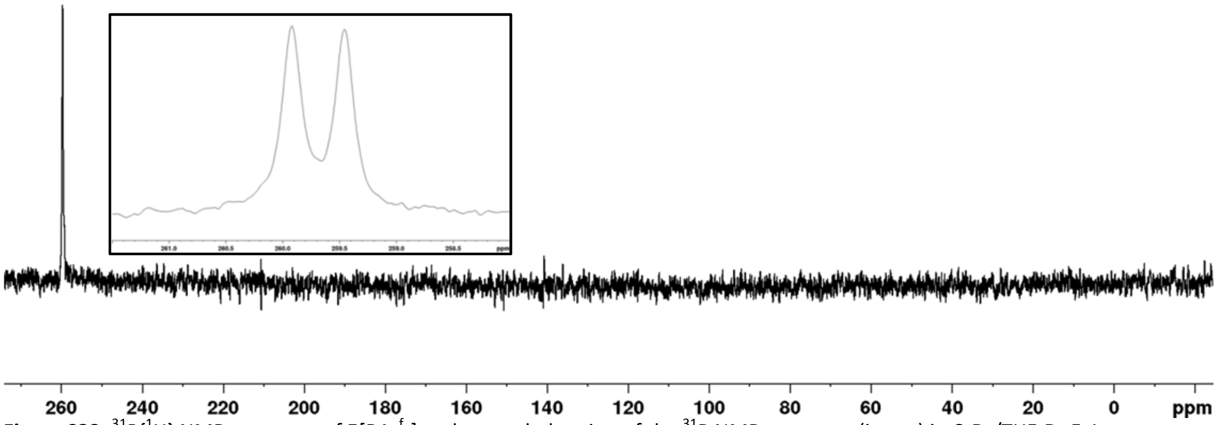

Figure S32. $^{31}P\{^{1}H\}$ NMR spectrum of **5**[BAr$^{f}_{4}$] and expanded region of the ^{31}P NMR spectrum (insert) in C_6D_6/THF-D_8 5:1.

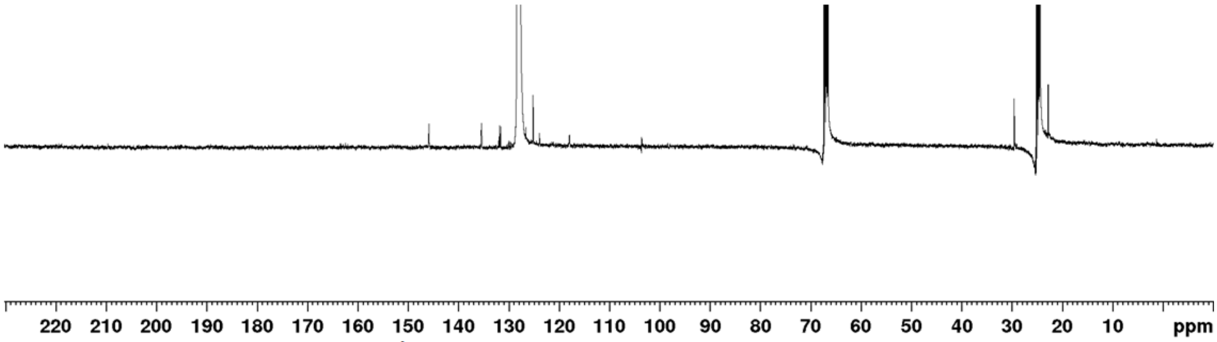

Figure S33. $^{13}C\{^1H\}$ NMR spectrum of **5**[BAr^f_4] in C_6D_6/THF-D_8 5:1.

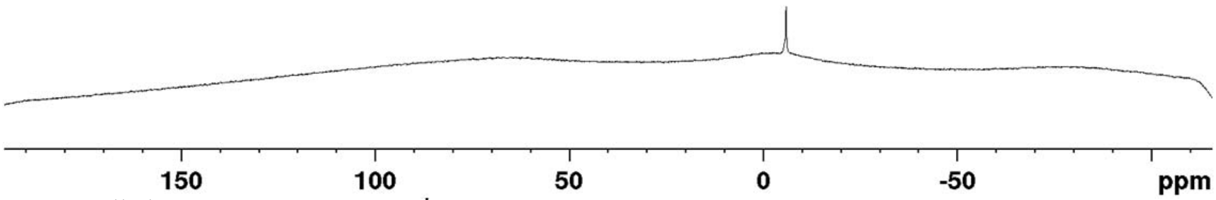

Figure S34. $^{11}B\{^1H\}$ NMR spectrum of of **5**[BAr^f_4] in C_6D_6/THF-D_8 5:1.

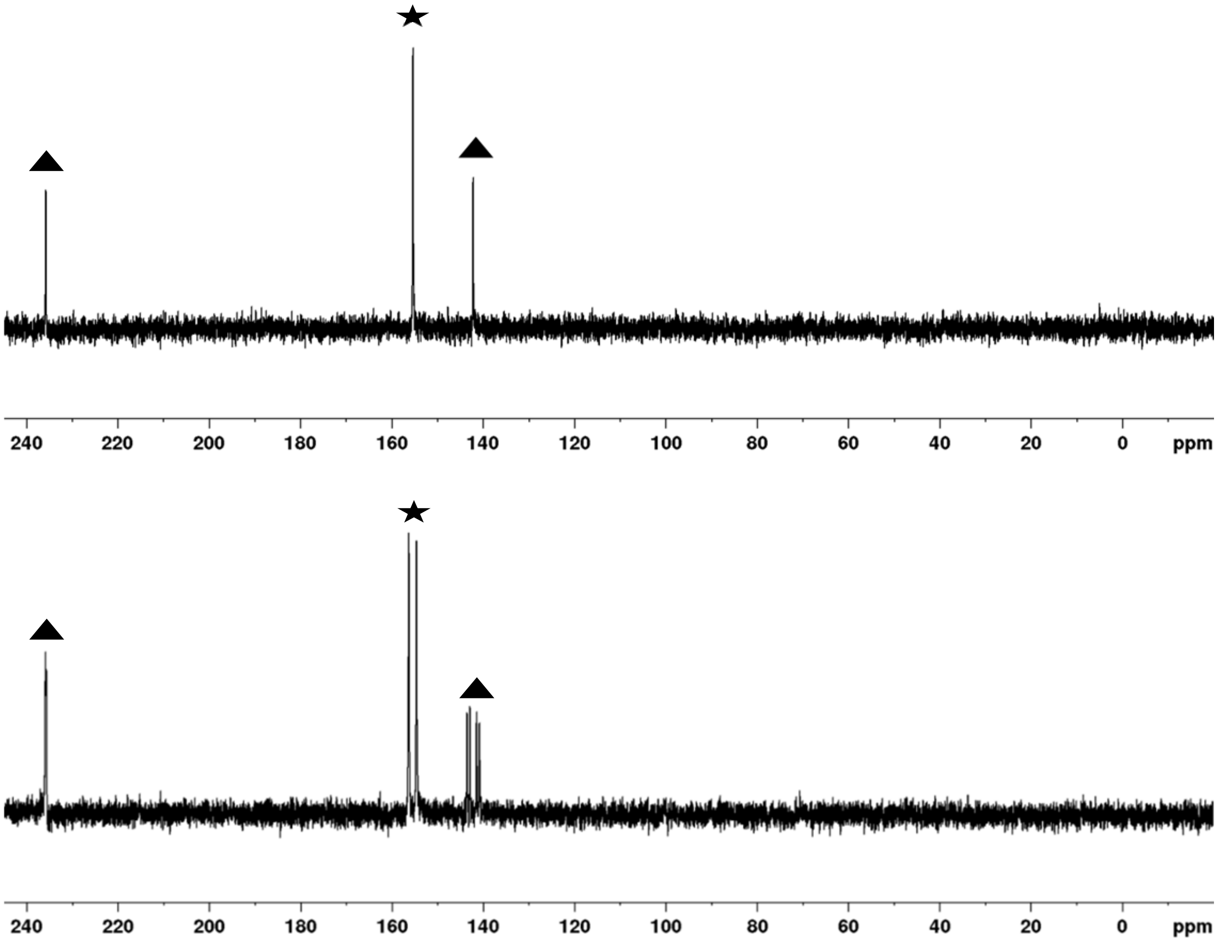

Figure S35. $^{31}P\{^1H\}$ (top) and ^{31}P NMR Spectrum of a solution of **2** in C_6D_6/CD_3CN (1:6) showing signals attributable to **2** (triangles) and **3b** (star).

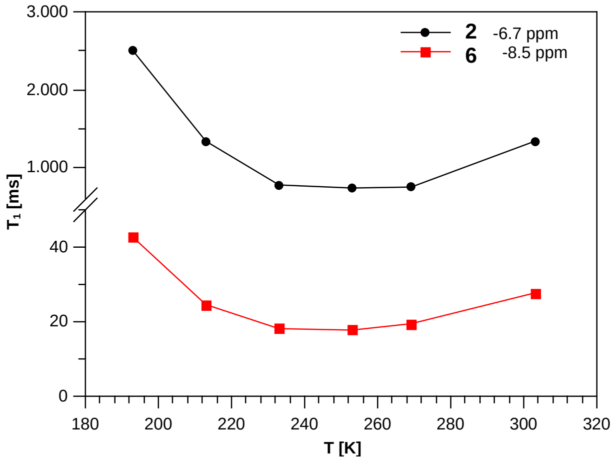

Figure S36. T_1 relaxation times for the hydride signals of **2** and **6** determined by the inversion-recvery method at different temperatures. Minimum values (at 253 K): $T_{1,min}$ = 18 ms (**6**), 739 ms (**2**).

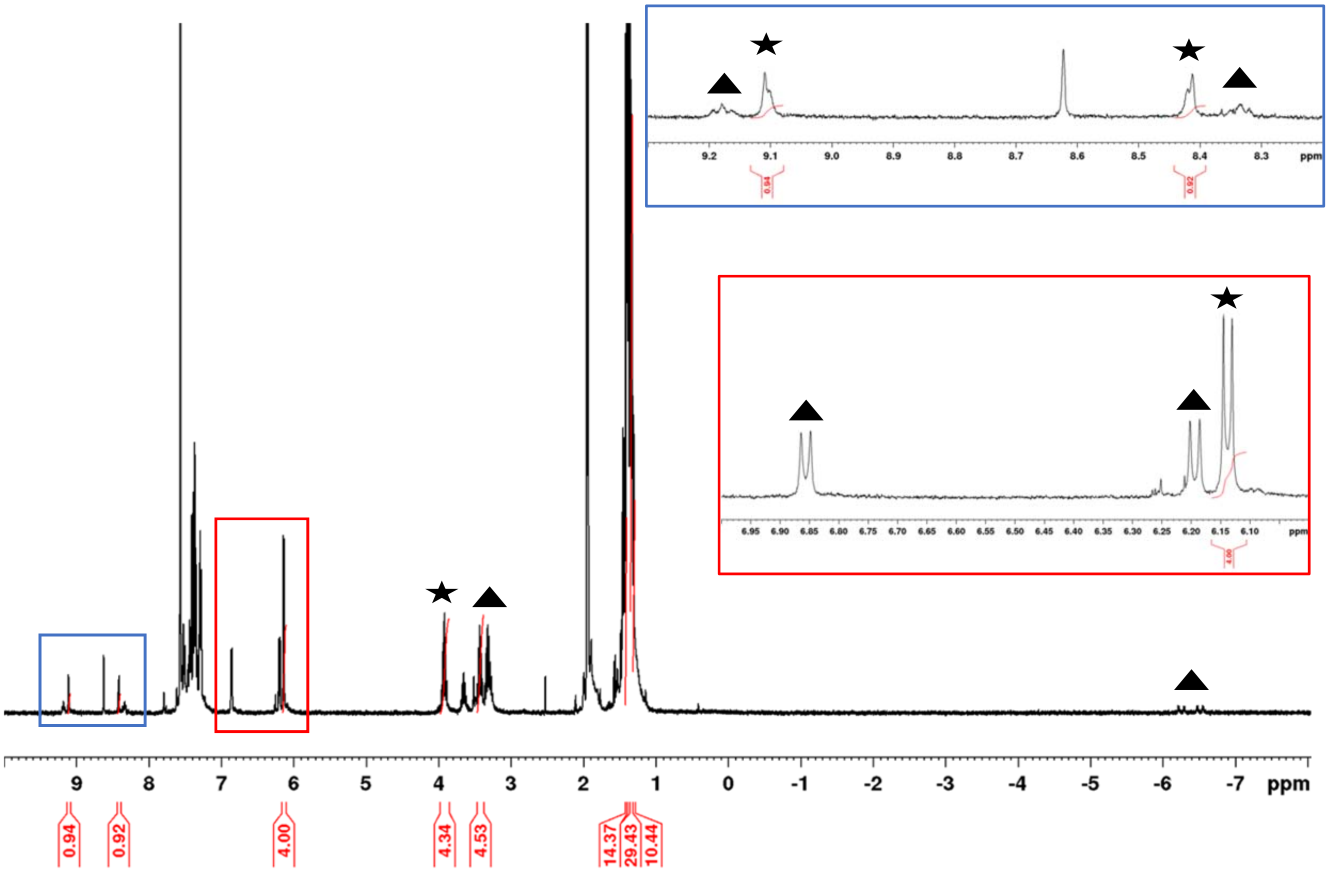

Figure S37. ^{1}H NMR Spectrum of a solution of **2** in C_6D_6/CD_3CN (1:6) showing signals attributable to **2** (triangles) and **3b** (star). The inserts display expansions of the regions containing the PH and NCH signals.

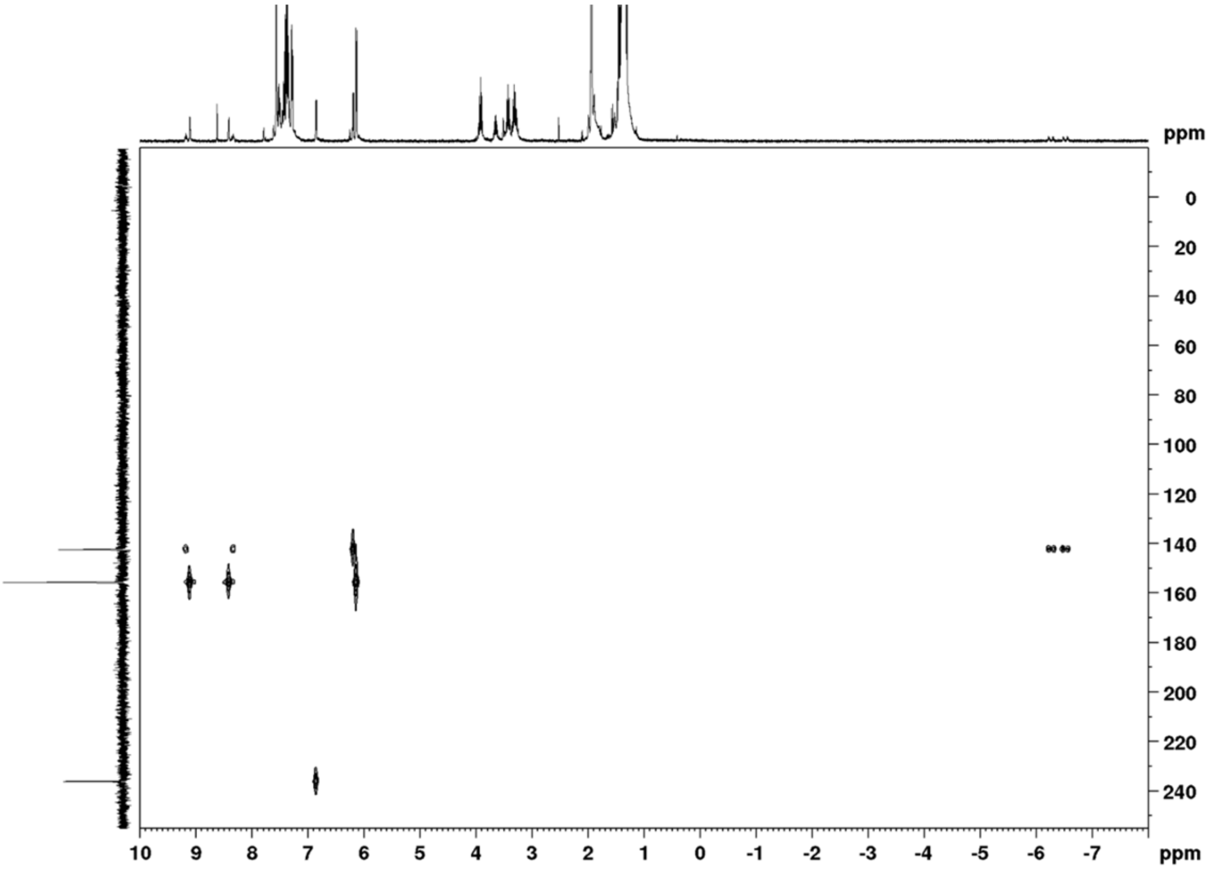

Figure S38. $^{1}H,^{31}P$ HMQC NMR spectrum of a solution of **2** in C_6D_6/CD_3CN (1:6).

High Resolution Mass Spectra

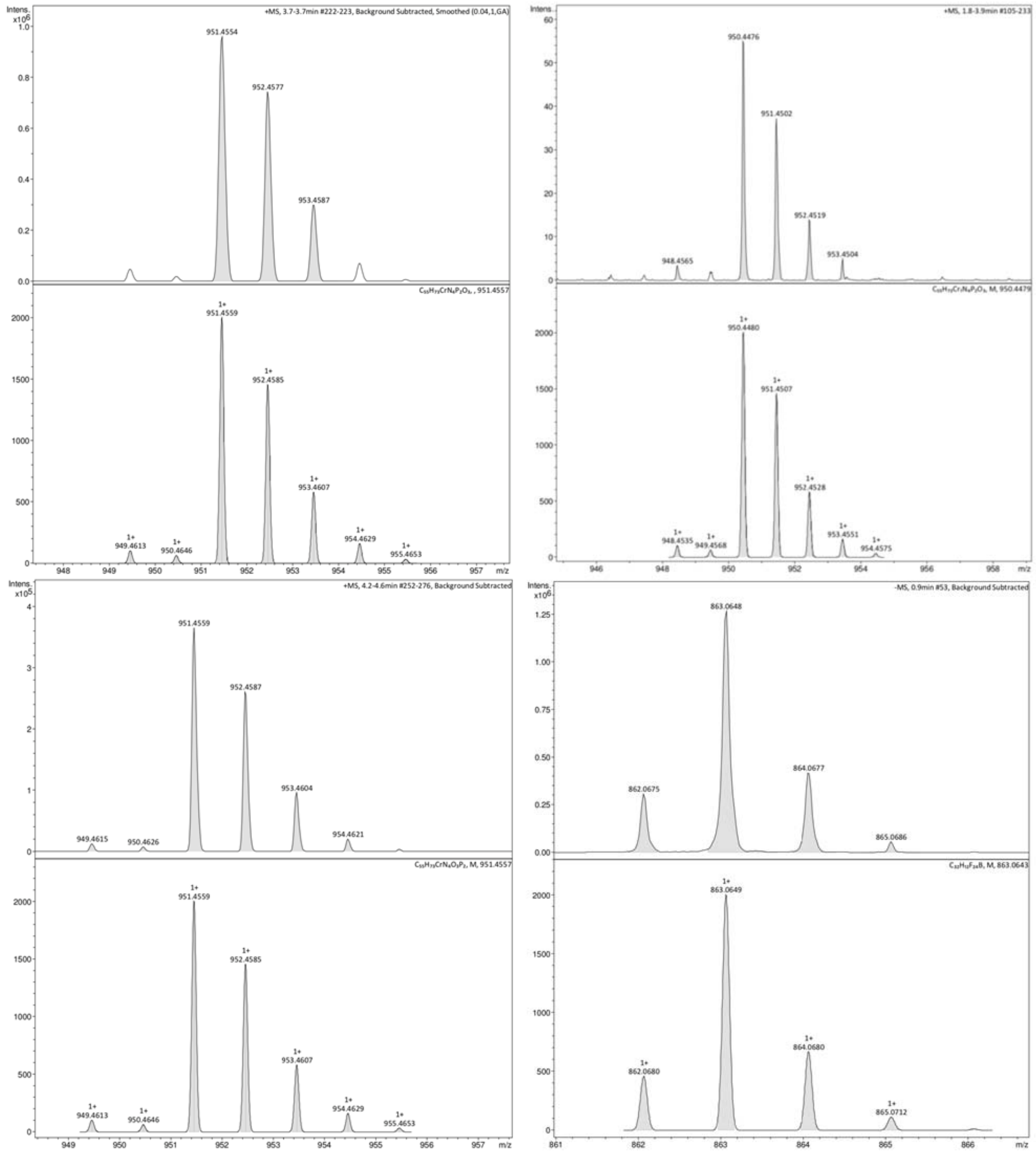

Figure S39. (+)-ESI-HRMS of **2** (top left), **4** (top right) and **5**[BAr$^f_4$] (bottom left) and (-)-ESI-HRMS of **5**[BAr$^f_4$] (bottom right). The top trace in each quadrant displays the observed spectrum and the bottom trace the simulated isotope pattern.

UV-VIS-Spectra

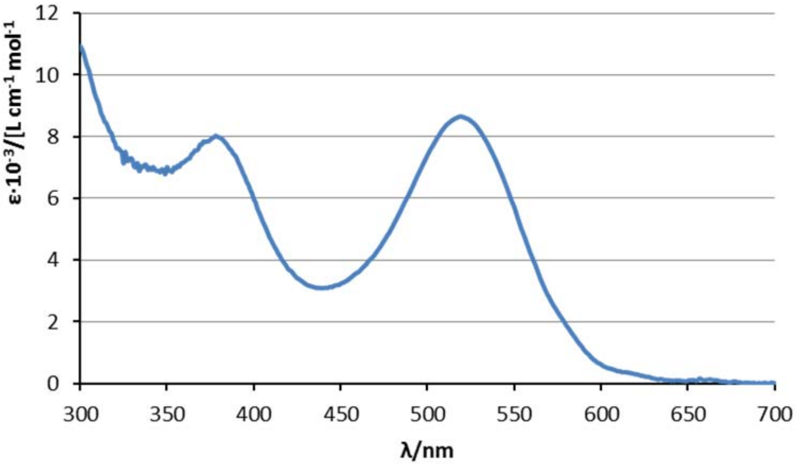

Figure S40: UV-VIS spectrum of **4** (0.02 mM in THF).

Crystallographic Studies

X-ray diffraction data were collected on a Bruker diffractometer equipped with a Kappa Apex II Duo CCD-detector and a KRYO-FLEX cooling device with Mo-K_α radiation (λ = 0.71073 Å) at 130(2) K for **2/3b** (co-crystal containing both molecules in 1:1 ratio) and with Cu-K_α radiation (λ = 1.5406 Å) at 135(2) K for **5**[BAr^f_4]. The structures were solved with direct methods (SHELXS-97[7]) and refined with a full-matrix least squares scheme on F^2 (SHELXL-2014 and SHELXL-97[7]). Semi-empirical absorption corrections were applied. Non-hydrogen atoms were refined anisotropically and hydrogens atoms using a riding model. The three carbonyl and one hydride ligands in **2** are disordered on four positions around the chromium atoms. Refinement under application of SIMU and SUMP with free occupation on all four positions at Cr gave three CO ligands displaced on four positions. The position of the hydrogen atom on the metal could not be refined. However, the presence of significantly different P-Cr distances, different coordination environments on the phosphorus atoms and a freely refined hydrogen atom on the pyramidal phosphorous atom, as well as conclusive NMR data provide proof for a phosphenium phosphane hydride complex with a metal bound hydrogen atom. In general, the data of the **2/3b** co-crystal are very weak (R_{int} = 27.9 %). In the crystal of **5**[BAr^f_4], the fluorine atoms on three CF_3-substituents in the anion displayed large anisotropic displacement parameters, and a refinement of disorder was performed by using split positions for each atom and refining the occupation of both positions with isotropic displacement parameters. The occupancies gained were then fixed and used for the anisotropic refinement of the disordered CF_3 groups. SADI was applied for C-F, F-F and 1,3-C-F distances. RIGU was applied for the displacement parameters, and ISOR for the disordered F atoms. CCDC-2005812 (**2/3b**) and CCDC-2005814 (**5**[BAr^f_4]) contain the crystallographic data for this paper, which can be obtained free of charge from the Cambridge Crystallographic Data Centre via www.ccdc.cam.ac.uk/data_request/cif.

Table S9. Crystallographic data of **2/3b** and **5**[BAr^f_4]

	**2/3b**	**5**[BAr^f_4]
Empirical formula	$C_{112}H_{151}Cr_2N_9O_6P_4$	$C_{87}H_{85}BCrF_{24}N_4O_3P_2$
Formula weight (g·mol^{-1})	1947.29	1815.33
T (K)	130(2)	135(2)
a [Å]	25.6854(10)	12.5935(3)
b [Å]	20.8290(9)	16.7577(4)
c [Å]	40.6993(18)	21.3290(5)
α [°]	90	95.414(1)
β [°]	90	99.551(1)
γ [°]	90	92.236(1)
V [Å^3]	21774.2(16)	4412.18(18)
Crystal system	Orthorhombic	Triclinic
Space group	Pbca	P-1
Z	8	2
ρ_{calc} [g·cm^{-3}]	1.188	1.366
μ [mm^{-1}]	0.314	2.291
Crystal dimensions [mm]	0.122 x 0.121 x 0.081	0.167 x 0.127 x 0.097
F(000)	8336	1868
R_{int}	0.2791	0.0456
Observed reflections [I>2σ(I)]	170081	65546
No of unique reflections	19979	15033
Restraints	13	1584
Parameters	1190	1087
R_1 [I>2σ(I)]	0.0827	0.0434
wR_2 (all data)	0.2301	0.1126
Goodness-of-fit on F^2	0.979	1.023
Largest diff. peak and hole [e$^-$ Å^{-3}]	0.776 and -0.411	0.456 and -0.390

Computational Studies

General remarks. All computations were performed with the Gaussian 16 program package.[8] DFT calculations were carried out using the ωB97xD[9] functional that had previously been successfully applied for the study of hydrogenation of NHP complexes,[10] using an ultrafine grid for numerical integration and Weigend's and Ahlrichs' def2-tzvp basis sets.[11] The molecular structures were established by full energy optimization. Subsequent stability tests (keyword stable=opt) confirmed that the electronic states are not compromised by singlet/triplet or RHF/UHF instabilities. Harmonic vibrational frequency calculations were finally carried out at the same level to establish the nature of the stationary points obtained as local minima (only positive normal modes) or transition states (one imaginary normal mode), and to calculate standard Gibbs free energies ΔG_0 (referring to p=1 bar and T=298K). NBO population analyses were carried out using the NBO module implemented in the Gaussian package. The electronic spectrum of $\mathbf{4^{Me}}$ was computed at the TD-ωB97xD/def2-tzvp level of theory. The relaxed molecular structure of the excited state was located by energy optimization of the first excited state identified in the TD-DFT calculation at the ωB97xD/def2-svp level of theory, and its energy recalculated at the final geometry at the ωB97xD/def2-tzvp level.

Table S10. Computed energies (in Hartree/particle) and atomic coordinates (in Å) for data of **2^{Me,Ph}, 4^{Me,Ph}**, **4^{Me}***, **6^{Me,Ph}, TS1**, **TS2**

Complex 2^{Me}

Charge = 0, Multiplicity = 1
E(RωB97xD) = -2601.98425927
Zero-point correction = 0.293092 (Hartree/Particle)
Thermal correction to Energy = 0.318439
Thermal correction to Enthalpy = 0.319383
Thermal correction to Gibbs Free Energy = 0.235454

C	0.0891497577	1.6866654266	1.1773628587
Cr	0.0431447163	0.009245362	0.3252289913
P	2.0907755914	0.0082433273	-0.0158867054
N	3.3243512019	-1.1217052234	-0.0156268408
C	4.5432656297	-0.6291946085	-0.4481496052
C	4.4579226815	0.6706667439	-0.7618400377
N	3.1729901513	1.1513588517	-0.5801945444
C	2.8035379448	2.5219258431	-0.880742159
O	0.1171334566	2.7136242827	1.6832628693
P	-2.0814651655	0.4546403917	-0.1916492711
N	-3.4462137915	-0.0928539113	0.6709322576
C	-4.5417952972	-0.1531791119	-0.2019494854
C	-4.1682514922	-0.1612706172	-1.4823839335
N	-2.7729573279	-0.1004466138	-1.640093517
C	-2.2062297843	0.1902066366	-2.9353772886
C	-0.156363648	-1.6163774011	-0.5949322765
O	-0.2601966923	-2.613654829	-1.1482785378
C	-0.1178451531	-0.8987632512	1.9383566323
O	-0.2227683693	-1.4661903709	2.9318792204
C	3.1457419393	-2.4981850197	0.4059754553
C	-3.6262889874	0.2760039721	2.0547962586
H	5.4093259787	-1.2681001968	-0.4981166462
H	5.2408725065	1.3189015211	-1.1188600746
H	-5.5418168696	-0.2687334802	0.1850004323
H	-4.8012229903	-0.292135091	-2.3452800802
H	-2.3275179091	1.2439565922	-3.2196690423
H	-1.1434913033	-0.0441967423	-2.9312241028
H	-2.6926663161	-0.4318675446	-3.6882601176
H	-4.4144940366	-0.3337114218	2.4990249486
H	-2.7052363917	0.085212432	2.6054550678
H	-3.8928906573	1.3345525595	2.1770750952
H	3.2612962886	2.8276418238	-1.8218331534
H	1.7208745187	2.5784137077	-0.9829181641
H	3.1213210938	3.1982914582	-0.0858503991
H	3.9098695587	-2.7677951702	1.1358372026
H	2.1665161107	-2.6004402862	0.8704114547
H	3.2002522786	-3.1767119209	-0.4465557151
H	-2.3671886878	1.8667985669	-0.2610182249
H	-0.122471534	0.870938313	-1.0773718222

Complex 6^{Me}

Charge = 0, Multiplicity = 1
E(RωB97xD) = -2601.94905120
Zero-point correction = 0.293003 (Hartree/Particle)
Thermal correction to Energy = 0.318743
Thermal correction to Enthalpy = 0.319687
Thermal correction to Gibbs Free Energy = 0.235769

C	0.3139816243	1.2912263786	0.5514594432
O	0.4316564996	2.2312117734	1.1970603011
Cr	0.0547533449	-0.2615571197	-0.4889982026
C	0.1529161342	-1.2955617756	1.0375137447
O	0.2276531792	-1.9261595477	1.9966099115
P	2.6290435703	-0.815479007	-0.3878250315
N	3.3108155335	0.0136385725	-1.7280423659
C	3.0523537685	-0.3912321831	-3.0829930464
P	-2.0048826104	-0.1788132045	-0.5088256884
N	-3.225784162	-0.476202464	-1.6205760433
C	-2.981615091	-0.8141445208	-3.0080045217
C	0.1822256399	-1.7722408314	-1.6030338609
O	0.268768034	-2.6998638264	-2.2719194981
N	3.3271197089	0.3805590196	0.6179642325
C	3.1817530008	0.3516004777	2.0503379047
C	3.5877323066	1.5496449434	-0.0867550474
C	3.5724845366	1.3486591855	-1.4103603764
N	-3.1819704087	0.0767752582	0.6580103397
C	-2.892484108	0.4057765778	2.0404275338
C	-4.4798981011	-0.0767556188	0.2015262273
C	-4.5056611136	-0.3946217237	-1.0988142752
H	3.8190502229	2.4660465768	0.432895773
H	3.8042714753	2.0636034901	-2.1839287126
H	-5.315976276	0.0576692284	0.8677569287
H	-5.3668426363	-0.5761500575	-1.7203637999
H	-3.5372244256	-0.1427541922	-3.6645023804
H	-1.918599261	-0.707143405	-3.2160243264
H	-3.2721723735	-1.8455085624	-3.2144230262
H	-3.2887895148	-0.362543589	2.705851291
H	-1.8148765264	0.4578687513	2.1763921426
H	-3.325429893	1.3724146189	2.3022067723
H	3.0760542671	-1.4793725486	-3.152099445
H	2.0728758494	-0.0498947604	-3.4474757857
H	3.8225710552	0.0077380428	-3.7456075795
H	2.1872785648	0.6694994554	2.3827119068
H	3.35218275	-0.6630653111	2.4129560124
H	3.9245231674	1.0058913017	2.5090744357
H	0.0355329373	0.7248916042	-1.9329284276
H	0.8363283452	0.7075739947	-1.6983574569

Complex 4^{Me}

Charge = 0, Multiplicity = 1
E(RωB97xD) = -2600.79353045
Zero-point correction = 0.277936 (Hartree/Particle)
Thermal correction to Energy = 0.302495
Thermal correction to Enthalpy = 0.303439
Thermal correction to Gibbs Free Energy = 0.221393

C	-0.1758589569	1.8608185516	0.85 60132016
Cr	0.0000053169	-0.0000908121	0.769682554
P	1.8324113505	0.17 80072904	-0.1911621389
N	3.2548890604	-0.7197930654	-0.2623348704
C	4. 2176647274	-0.1879839482	-1.1048139214

Complex 4^{Me}* (1st excited state, optimized)

Charge = 0, Multiplicity = 1
E(RωB97xD) [a] = -2600.71274853 (-2599.35187811)
Zero-point correction [b] = 0.277385 (Hartree/Particle)
Thermal correction to Energy [b] = 0.302223
Thermal correction to Enthalpy [b] = 0.303167
Thermal correction to Gibbs Free Energy [b] = 0.219695

C	0.3918423264	1.0628632522	0.3086714733
Cr	0.1175338914	-0.6169184833	-0.436483527
P	2.455651175	-0.595986516	-0.6536795292
N	3.3475409232	0.071985576	0.6674917347
C	4.0271446472	1.2236397534	0.3416989439

C	3.8036369432	0.9556033631	-1. 6640097521		C	3.8647664323	1.574489764	-0.9579127551
N	2.5170259691	1.2791606882	-1.2660009453		N	3.0395081107	0.7040941257	-1.635776954
C	1.8086671743	2. 4313162634	-1.7812030464		C	2.6732114497	0.8669922021	-3.0220354328
O	-0.2695265898	3.0062302448	0.9246141794		O	0.5984095047	2.1031937794	0.7752733986
P	- 1.832446128	-0.1779614996	-0.1911256987		P	-1.9574668779	-0.3564138599	-0.541801082
N	-3.2548741482	0.7199337419	- 0.262142705		N	-3.080147841	0.5300777077	0.3800585089
C	-4.2176982613	0.1882969894	-1.1046766422		C	-4.3893648653	0.4135521106	-0.0592416822
C	-3.8037525261	-0.9552394903	-1.6640356765		C	-4.4870609177	-0.4066670823	-1.126983983
N	-2.5171517757	-1.2789303374	-1.26609950 45		N	-3.2483842031	-0.8979120217	-1.5113813302
C	-1.8088750934	-2.43106238	-1.7814663597		C	-3.0742837277	-1.8042076653	-2.6286050858
C	0.175890317	-1.86101825 78	0.8555924157		C	0.1315885356	-2.1236623012	-1.557524775
O	0.2696035216	-3.0064450801	0.9238731832		O	0.14697793	-3.0599604827	-2.2348229104
C	0.00006014 2	-0.0003017946	2.6285906754		C	0.1229437939	-1.5266395019	1.1330347263
O	0.0002559845	-0.0004947406	3.7752623236		O	0.1046636291	-2.0904447612	2.1463946129
C	3.4894404296	-1.9061740604	0.5340405929		C	3.2135312508	-0.4034878803	2.0258289794
C	-3.489339378	1.9062206545	0.5343973007		C	-2.6998574694	1.3212222478	1.5331998429
H	5.1652317778	-0.6832349901	-1.2378960896		H	4.621411126	1.7456285205	1.0897102392
H	4.343878461 2	1.5902925831	-2.3468164155		H	4.3089834247	2.4291065077	-1.4650368159
H	-5.1652384296	0.6836228188	-1.237671596 7		H	-5.1939822969	0.9427518652	0.4468923136
H	-4.3440483218	-1.5898036287	-2.3469156151		H	-5.3876180214	-0.6854845505	-1.6700676503
H	-2.1062022312	-2.61092 19827	-2.8150846892		H	-3.8631483086	-1.6273451095	-3.3729043683
H	-0.7376070172	-2.2303072362	-1.7564155339		H	-2.1022499784	-1.6228495199	-3.1066800399
H	-2.0 066849057	-3.3259066318	-1.1884300009		H	-3.108988837	-2.8583193795	-2.3121694391
H	-4.5381677739	1.9457997972	0.8 296013849		H	-3.5311756565	1.3499307088	2.2512591351
H	-2.8769750756	1.860775593	1.4344398825		H	-1.8326373055	0.8591272348	2.0245092325
H	-3.2340863158	2.8 170906889	-0.0106187567		H	-2.4279251468	2.3510168651	1.2536365514
H	2.1058600071	2.6112514855	-2.8148473056		H	3.4264025491	0.4397879858	-3.7049798519
H	0. 7374070093	2.2305369943	-1.7560286789		H	1.7094905386	0.3665164486	-3.1988204717
H	2.0065368207	3.3261220016	-1.1 881288938		H	2.544988423	1.9339063941	-3.2571657249
H	4.5382564693	-1.9456851457	0.8292984415		H	2.4151623428	0.1341232215	2.5639731259
H	2.8770189179	-1. 8609273028	1.4340538371		H	2.959209965	-1.4716597907	2.0267959924
H	3.2343144917	-2.816988383	-0.0111277307		H	4.1628504836	-0.274235365	2.5660485978

[a] at the RωB97xD/def2-tzvp//RωB97xD/def2-svp level (RωB97xD/def2-svp energy in parentheses). [b] at the RωB97xD/def2-svp level

**TS 2**					**TS 1**			
Charge = 0, Multiplicity = 1<br>E(RωB97xD) = -2601.8926063<br>Zero-point correction = 0.288136 (Hartree/Particle)<br>Thermal correction to Energy = 0.313627<br>Thermal correction to Enthalpy = 0.314571<br>Thermal correction to Gibbs Free = 0.230205					Charge = 0, Multiplicity = 1<br>E(RωB97xD) = -2601.92791598<br>Zero-point correction = 0.289114 (Hartree/Particle)<br>Thermal correction to Energy = 0.315668<br>Thermal correction to Enthalpy = 0.316612<br>Thermal correction to Gibbs Free Energy = 0.231190			
C	0.0308874726	1.6503278852	0.9303843371		C	0.4096173619	1.1187311003	0.7381072281
Cr	0.0753980225	0.2395898728	-0.3094634551		O	0.5933361448	1.9051879458	1.5504699688
P	2.1543397541	0.1165139852	-0.197823872		Cr	0.1176225852	-0.2514889171	-0.5322077336
N	3.2457930276	-1.1501130675	-0.1028965594		C	-0.1191058449	-1.4053326358	0.8486232921
C	4.5538290309	-0.7541918145	0.1158467107		O	-0.2573232385	-2.1331611208	1.7320962068
C	4.6546497327	0.5790692059	0.1998979212		P	2.4853877482	-0.9198232872	-0.3799460818
N	3.4227231762	1.1856998327	0.0309294882		N	2.8037537577	0.1984896325	-1.6575215134
C	3.2552382559	2.6267677065	0.0481943872		C	2.9225166529	-0.1524508838	-3.0508950709
O	0.0379667151	2.5157798124	1.683780772		P	-1.9467447584	-0.0030739625	-0.6828039337
P	-2.2377939067	0.6359283425	-0.6522985982		N	-3.1716031565	-0.7241251682	-1.5846008291
N	-3.3754185989	0.5969473019	0.6261806311		C	-2.9661666398	-1.3470092676	-2.87502814
C	-4.3790896439	-0.3433630355	0.3621468534		C	0.1354165835	-1.7063894871	-1.719459508
C	-4.2953725976	-0.8894664856	-0.8486659825		O	0.1507825301	-2.6388446272	-2.388610879
N	-3.1760742034	-0.4619065081	-1.5806624889		N	3.3147466966	0.1494166157	0.6899546696
C	-3.2706208326	-0.30414124	-3.014280422		C	3.3656120595	-0.0920705683	2.1083561302
C	-0.0873174012	-1.0425483194	-1.6756210609		C	3.5376693906	1.3959352471	0.1293149985
O	-0.1890913522	-1.8460378324	-2.4852550777		C	3.2930487246	1.4214121733	-1.1857468417
C	-0.1885852914	-1.0633043448	0.971774539		N	-3.0994121024	0.4199499295	0.4714000636
O	-0.395003492	-1.8695024493	1.7642640476		C	-2.7678172088	1.0855173238	1.7161134426

C	2.8777002069	-2.5493246636	-0.2124440436		C	-4.3693428908	-0.0615634878	0.2011437816
C	-3.0710573085	1.0273666725	1.9668967963		C	-4.4131249238	-0.7130327789	-0.9666256379
H	5.3410319299	-1.4850545988	0.1997412506		H	3.8749181005	2.2194547661	0.7398699633
H	5.5408217419	1.1677249334	0.3704061819		H	3.4406881036	2.2480933857	-1.8630558278
H	-5.1139831549	-0.5605601564	1.1214515764		H	-5.1798564042	0.1152511879	0.8886778037
H	-4.965500936	-1.6180352963	-1.2775417162		H	-5.2652673664	-1.1775798217	-1.4342279387
H	-3.8784560903	0.5636209818	-3.3024099785		H	-3.7855794724	-1.0860097629	-3.5461821142
H	-2.2756132256	-0.1857644306	-3.4432990143		H	-2.0361333754	-0.9829320316	-3.3079770927
H	-3.7102419042	-1.2027993569	-3.4508809738		H	-2.9009945947	-2.4335630224	-2.7896210532
H	-2.335028397	0.3803746512	2.4593081493		H	-2.2133045661	0.4232375868	2.3844077937
H	-2.6825855712	2.0464773404	1.9637838897		H	-2.1648784123	1.9720101387	1.5269307119
H	-3.9861236204	1.0246275823	2.5604918854		H	-3.6878751797	1.3955597561	2.2094041798
H	3.9978715188	3.0945611361	-0.5990298675		H	2.5001986876	-1.1419883246	-3.2202987268
H	2.2627643663	2.872076983	-0.3261742982		H	2.3745113528	0.5604382497	-3.6757451263
H	3.3548783241	3.020664973	1.060682631		H	3.9678878365	-0.1682953835	-3.3777802479
H	3.1939976515	-3.0934665939	0.6783023794		H	2.4255118711	0.1603141775	2.612940137
H	1.7968078936	-2.6285868673	-0.3011618516		H	3.5760431637	-1.147682683	2.2904504072
H	3.3351033301	-2.9983406372	-1.0952239885		H	4.1676713102	0.4963232659	2.5570338789
H	-1.1941358832	1.6295479672	-1.4167707813		H	0.1913127167	1.4658023669	-2.2874641641
H	-0.1157377395	1.5446165322	-1.5360253972		H	0.8079937569	1.7885143729	-2.0005961966

4^{Ph}					2^{Ph}			
Charge = 0, Multiplicity = 1<br>E(RωB97xD) = -3367.73710269					Charge = 0, Multiplicity = 1<br>E(RωB97xD) = -3368.92928821			
C	16.7704510431	7.0582903654	12.8946427834		C	-5.905728	1.136123	-0.761136
O	15.6913371439	6.8323218738	12.5629079194		C	-5.065475	0.465898	0.126091
Cr	18.5119812944	7.2686392704	13.54350267		C	-3.810488	0.982556	0.435751
C	18.3002325361	5.4981526626	14.0459712669		C	-3.39458	2.174845	-0.159197
O	18.1685946297	4.3981000939	14.3581327884		C	-4.226673	2.846397	-1.057527
P	17.9329528923	8.8430902588	14.7665976877		C	-5.485249	2.327826	-1.350707
N	16.6460648394	9.9739033383	14.6724811539		N	-2.111877	2.71925	0.154202
C	15.6320602458	9.9212043071	13.6743434796		C	-1.959231	3.991483	0.702381
P	19.3438859424	7.8096692027	11.7199533274		C	-0.666539	4.268588	0.961524
N	20.8498455693	8.5124515947	11.2935016177		N	0.165683	3.217533	0.58991
C	21.887863726	8.7649887986	12.2347752919		C	1.568404	3.200416	0.857208
C	20.2023799638	7.0505625999	14.3137214826		C	2.473578	2.960403	-0.177129
O	21.224544478	6.7998027712	14.7805118691		C	3.831196	2.846632	0.110103
N	18.5287314792	9.7382626607	16.1114404076		C	4.288846	3.003839	1.416842
C	19.7018814907	9.4163373931	16.8411488223		C	3.383498	3.274831	2.442149
C	17.7576942747	10.8572832575	16.4370431732		C	2.021322	3.361023	2.168263
C	16.6882201845	10.9803325262	15.6325041294		H	2.116673	2.844022	-1.200366
N	18.8757394834	7.9821103328	10.0719559432		H	1.300588	3.521287	2.972875
C	17.6178256058	7.5932079025	9.5439087977		H	-3.143785	0.450623	1.116519
C	19.850718725	8.5699908849	9.2617004998		H	-3.878106	3.766525	-1.531412
C	20.9689984128	8.855615072	9.9502321906		P	-0.655311	1.876534	-0.05948
H	18.0317344502	11.4824750718	17.2832320762		Cr	-0.05618	-0.02685	-0.64176
H	15.9120712859	11.7417193094	15.6606100659		P	0.465171	-2.220356	-0.85522
H	19.667812331	8.7077885771	8.1988941775		N	-0.24638	-3.408062	0.192536
H	21.8893115167	9.3011284026	9.579492507		C	-1.541994	-3.334401	0.732772
C	20.638897178	10.4159904168	17.1262472682		C	-1.756695	-3.511689	2.105509
C	21.7822355192	10.1103543155	17.8597587817		C	-3.046745	-3.448947	2.626554
C	22.0102297563	8.80517808	18.2937202748		C	-4.135329	-3.192039	1.793029
C	21.0760261438	7.8113744096	18.0073193646		C	-3.921686	-3.009688	0.426661
C	19.9175578389	8.1141258423	17.2982107426		C	-2.637841	-3.089412	-0.102977
H	20.4727761757	11.4311187145	16.7585890955		H	-0.902066	-3.67547	2.765093
H	22.5078414036	10.8967209049	18.0798056248		H	-2.484928	-2.96748	-1.176155
H	22.9145180047	8.5632364961	18.85546234		C	-1.573744	-0.328957	-1.676711
H	21.2454896182	6.7862519871	18.3418614798		O	-2.528643	-0.543516	-2.283304
H	19.180961159	7.3397002274	17.0782940979		H	-1.010935	-0.651965	0.515732
C	14.3124766967	9.6477290168	14.0381273872		C	1.149083	-0.037195	0.789685

C	13.3256659674	9.5740500284	13.0582330811		O	1.80184	-0.07492	1.734861
C	13.6572224056	9.7575211232	11.7168669406		C	1.039867	0.514911	-2.022217
C	14.9757690668	10.0313719923	11.3575291515		O	1.686163	0.916208	-2.894356
C	15.9638520978	10.1222261003	12.3329860989		N	2.048063	-2.809245	-0.447766
H	14.0729597117	9.4729614051	15.0890500374		C	3.24013	-2.066464	-0.562324
H	12.2953874224	9.3519598519	13.3436607079		C	4.126373	-1.965264	0.51819
H	12.8870215865	9.6786598972	10.9470203339		C	5.292521	-1.215819	0.397989
H	15.2400956538	10.1641345615	10.3074949105		C	5.581591	-0.541216	-0.788476
H	17.0029340711	10.3177879374	12.0630762146		C	4.700699	-0.640267	-1.864073
C	23.0908971171	8.0624481807	12.1476577296		C	3.547823	-1.413642	-1.760084
C	24.0967349928	8.2940957649	13.082561289		H	3.875372	-2.445808	1.465531
C	23.8970160527	9.2132939626	14.111173283		H	2.878049	-1.510325	-2.615643
C	22.6948037445	9.9136115599	14.1943687093		C	1.970753	-3.987543	0.318735
C	21.6917655597	9.698784859	13.2542385654		C	0.719786	-4.312935	0.668041
H	23.2208882712	7.3210222399	11.3566191056		H	0.259377	-2.835166	-2.13839
H	25.0340576969	7.7377711398	13.0177800069		H	2.876279	-4.556473	0.520051
H	24.6777137765	9.3790323359	14.856246396		H	0.401433	-5.201609	1.209526
H	22.5314095308	10.6252087196	15.0049226814		H	-0.245478	5.176922	1.385943
H	20.7391492813	10.227098278	13.3189718472		H	-2.83063	4.617041	0.880111
C	16.9053928332	8.4761630646	8.7245246927		H	5.970561	-1.139166	1.250908
C	15.6798449706	8.0922707687	8.1866274963		H	4.911453	-0.121133	-2.80136
C	15.1465762535	6.8377116132	8.4790195		H	-3.199827	-3.58727	3.699296
C	15.8576387315	5.9608711617	9.2963016495		H	-4.762286	-2.8174	-0.243737
C	17.0957687091	6.3264502123	9.8158474848		H	4.531918	2.625968	-0.696975
H	17.311154183	9.4699613429	8.5220905728		H	3.735651	3.393127	3.468803
H	15.1308500131	8.7865229429	7.5463822885		H	-5.382534	-0.469895	0.589304
H	14.1783644816	6.5434452294	8.0693843163		H	-6.135006	2.852153	-2.054287
H	15.448527939	4.9766838035	9.5319214939		H	6.491283	0.057042	-0.873849
H	17.6580318403	5.6396369847	10.45053083		H	-5.144854	-3.139887	2.206464
					H	5.353683	2.909059	1.638336
					H	-6.889611	0.726763	-0.998874

**6^{Ph}**			
Charge = 0, Multiplicity = 1<br>E(RωB97xD) = -3368.90711089			
C	0.347853171	1.3350594822	0.6215477565
Cr	0.7081575555	2.7388775647	1.8246710064
C	0.8163439369	3.9881138762	3.2408707096
O	0.9869416554	4.7247924339	4.1052815573
O	0.1657152683	0.4631141028	-0.1012403329
C	1.722325165	1.5678072898	2.8411808935
O	2.3530270954	0.85860194	3.4918893695
P	-1.321338747	1.9487648912	3.0902280498
N	-2.021419471	0.5934822083	2.1978036499
C	-2.8887952512	1.0318192696	1.1957210282
C	-3.2106171866	2.3310865841	1.3003951509
N	-2.6144551825	2.9620003522	2.397861208
C	-2.7017986549	4.3243355327	2.657804617
P	2.3342278991	3.5125387094	0.7977911246
N	3.8905169353	2.9777478449	0.38288627
C	4.6443040906	3.9029079055	-0.3338284191
C	3.9515106112	5.0366530405	-0.5615796582
N	2.6793186939	4.9781206902	-0.0001103568
C	1.6967553289	5.9963688196	-0.1771058644
C	-1.6447182285	-0.7299960018	2.3957478599
C	4.3964979979	1.7069631681	0.7935460193
H	-3.2406285709	0.3556929487	0.4210481654
H	-3.9042942484	2.8616661178	0.6536938909
H	5.6590013521	3.6603173301	-0.6405862056
H	4.27935886	5.9303313439	-1.0874286254

H	-0.1456363197	4.0116003257	0.9795002304
H	-0.6945850676	3.3882279008	1.048471058
C	-3.1771896418	5.2423888179	1.7015091949
C	-3.2316597739	6.6026163706	1.9919431702
C	-2.7972588789	7.092413997	3.2228818222
C	-2.3091672834	6.1900484885	4.1687950985
C	-2.2693894985	4.8273208903	3.8990319249
H	-3.4941210817	4.8989985668	0.7165507781
H	-3.6096043662	7.2908796386	1.2317490877
H	-2.8410205173	8.1608075819	3.4438977374
H	-1.9613986904	6.5477757227	5.1405292466
H	-1.9072157791	4.1317511509	4.6593172312
C	-0.5857449132	-1.0377138284	3.2695443015
C	-0.1817446331	-2.3545339791	3.4639922573
C	-0.8128373424	-3.4021368316	2.7961497358
C	-1.8709998147	-3.1057428574	1.9370749934
C	-2.2898427867	-1.7945812406	1.7382106787
H	-0.0769684123	-0.2403921969	3.8133177611
H	0.6459493505	-2.556036343	4.1479682417
H	-0.4905508876	-4.4341039176	2.9464879273
H	-2.3908001562	-3.9114212494	1.4128413857
H	-3.1352776376	-1.6049181308	1.0768894434
C	1.3199491577	6.3799730636	-1.4668653049
C	0.3236153044	7.338272999	-1.6374954433
C	-0.3143870475	7.8934779068	-0.5282380096
C	0.0626822884	7.5062275814	0.7563186948
C	1.0781040894	6.5700113986	0.9357727029
H	1.7963352187	5.9112848747	-2.3306746509
H	0.0303763802	7.6369552789	-2.6460001288
H	-1.1100141979	8.6283414067	-0.6658859005
H	-0.4387052262	7.9249456953	1.6305270747
H	1.3877573092	6.282967756	1.9408973316
C	3.7849574331	0.5380249406	0.3391534241
C	4.2402990173	-0.6968858056	0.7933890493
C	5.3154643376	-0.7653937468	1.6774154064
C	5.9356814446	0.4051994614	2.1129953849
C	5.4722162065	1.6448627806	1.6809001072
H	2.9472197776	0.5975700206	-0.35720495
H	3.7469350232	-1.6093810436	0.4539178607
H	5.6683741403	-1.7350284576	2.0337801771
H	6.7733314771	0.3551968806	2.8113431496
H	5.9257529224	2.5694656871	2.0441478544

References

1 V. Desorby, E. P. Kündig, *Helv. Chim. Acta* **1981**, *64*, 1288-1297

2 J. M. Maher, R. P. Beatty, N. J. Cooper, *Organometallics* **1985**, *4*, 1354-1361

3 S. Burck, D. Gudat, M. Nieger, W.-W. Du Mont, *J. Am. Chem. Soc.* **2006**, *128*, 3946-3955

4 M. Brookhart, B. Grant, A. F. Volpe, Jr., *Organometallics* **1992**, *11*, 3920-3922

5 R. H. Harris, E. D. Becher, S. M. Cabral de Menezes, R. Goodfellow P. Granger, *Concepts Magn. Reson*., 2002, **14**, 326–346.

6 M. Gediga, S. H. Schlindwein, J. Bender, M. Nieger and Dietrich Gudat, *Angew. Chem. Int. Ed.* **2017**, *56*, 15718-15722

7 G. M. Sheldrick, *Acta Cryst.* **2008**, *A64*, 112-122.

8 Gaussian 16, Revision C.01, M. J. Frisch, G. W. Trucks, H. B. Schlegel, G. E. Scuseria, M. A. Robb, J. R. Cheeseman, G. Scalmani, V. Barone, G. A. Petersson, H. Nakatsuji, X. Li, M. Caricato, A. V. Marenich, J. Bloino, B. G. Janesko, R. Gomperts, B. Mennucci, H. P. Hratchian, J. V. Ortiz, A. F. Izmaylov, J. L. Sonnenberg, D. Williams-Young, F. Ding, F. Lipparini, F. Egidi, J. Goings, B. Peng, A. Petrone, T. Henderson, D. Ranasinghe, V. G. Zakrzewski, J. Gao, N. Rega, G. Zheng, W. Liang, M. Hada, M. Ehara, K. Toyota, R. Fukuda, J. Hasegawa, M. Ishida, T. Nakajima, Y. Honda, O. Kitao, H. Nakai, T. Vreven, K. Throssell, J. A. Montgomery, Jr., J. E. Peralta, F. Ogliaro, M. J. Bearpark, J. J. Heyd, E. N. Brothers, K. N. Kudin, V. N. Staroverov, T. A. Keith, R. Kobayashi, J. Normand, K. Raghavachari, A. P. Rendell, J. C. Burant, S. S. Iyengar, J. Tomasi, M. Cossi, J. M. Millam, M. Klene, C. Adamo, R. Cammi, J. W. Ochterski, R. L. Martin, K. Morokuma, O. Farkas, J. B. Foresman, and D. J. Fox, Gaussian, Inc., Wallingford CT, 2016.

9 J.-D. Chai, M. Head-Gordon, *Phys. Chem. Chem. Phys.* **2008**, *10*, 6615–6620.

10 M. Gediga, C. M. Feil, S. H. Schlindwein, J. Bender, M. Nieger, D. Gudat, *Chem. Eur. J.* **2017**, *23*, 11560-11569.

11 F. Weigend R. Ahlrichs, *Phys. Chem. Chem. Phys.* ***2005***, *7*, 3297-3305.

Author Contributions

N. Birchall: experimental and spectroscopic studies, writing of original draft, crystal structure solution

C. M. Feil: crystal structure solution

M. Gediga: experimental studies (supporting)

M. Nieger: crystal structure solution

D. Gudat: spectroscopic studies (supporting), project supervision, writing of final manuscript, computational studies

All authors: correction of manuscript.
